# The Rsm (Csr) post-transcriptional regulatory pathway coordinately controls multiple CRISPR–Cas immune systems

**DOI:** 10.1093/nar/gkab704

**Published:** 2021-08-17

**Authors:** Aroa Rey Campa, Leah M Smith, Hannah G Hampton, Sahil Sharma, Simon A Jackson, Thorsten Bischler, Cynthia M Sharma, Peter C Fineran

**Affiliations:** Department of Microbiology and Immunology, University of Otago, PO Box 56, Dunedin 9054, New Zealand; Bio-Protection Research Centre, University of Otago, PO Box 56, Dunedin 9054, New Zealand; Department of Microbiology and Immunology, University of Otago, PO Box 56, Dunedin 9054, New Zealand; Department of Microbiology and Immunology, University of Otago, PO Box 56, Dunedin 9054, New Zealand; Chair of Molecular Infection Biology II, Institute of Molecular Infection Biology (IMIB), University of Würzburg, 97080 Würzburg, Germany; Department of Microbiology and Immunology, University of Otago, PO Box 56, Dunedin 9054, New Zealand; Genetics Otago, University of Otago, Dunedin, New Zealand; Core Unit Systems Medicine, University of Würzburg, 97080 Würzburg, Germany; Chair of Molecular Infection Biology II, Institute of Molecular Infection Biology (IMIB), University of Würzburg, 97080 Würzburg, Germany; Department of Microbiology and Immunology, University of Otago, PO Box 56, Dunedin 9054, New Zealand; Bio-Protection Research Centre, University of Otago, PO Box 56, Dunedin 9054, New Zealand; Genetics Otago, University of Otago, Dunedin, New Zealand

## Abstract

CRISPR–Cas systems provide bacteria with adaptive immunity against phages and plasmids; however, pathways regulating their activity are not well defined. We recently developed a high-throughput genome-wide method (SorTn-seq) and used this to uncover CRISPR–Cas regulators. Here, we demonstrate that the widespread Rsm/Csr pathway regulates the expression of multiple CRISPR–Cas systems in *Serratia* (type I-E, I-F and III-A). The main pathway component, RsmA (CsrA), is an RNA-binding post-transcriptional regulator of carbon utilisation, virulence and motility. RsmA binds *cas* mRNAs and suppresses type I and III CRISPR–Cas interference in addition to adaptation by type I systems. Coregulation of CRISPR–Cas and flagella by the Rsm pathway allows modulation of adaptive immunity when changes in receptor availability would alter susceptibility to flagella-tropic phages. Furthermore, we show that Rsm controls CRISPR–Cas in other genera, suggesting conservation of this regulatory strategy. Finally, we identify genes encoding RsmA homologues in phages, which have the potential to manipulate the physiology of host bacteria and might provide an anti-CRISPR activity.

## INTRODUCTION

Bacteria have multiple defence systems to protect themselves from invasion by phages and mobile genetic elements (MGEs) (1). Their adaptive immunity is provided by CRISPR–Cas (clustered regularly interspaced short palindromic repeats and CRISPR-associated proteins) systems, which enable protection in a sequence-specific manner (2,3). CRISPR–Cas defence has three phases: firstly, adaptation occurs when the cell encounters an invader. Here, a short segment of the invader nucleic acid gets incorporated into the CRISPR array as a ‘spacer’, separated from other spacers by short repeats (4,5). During biogenesis, CRISPR arrays are expressed and processed into guide CRISPR RNAs (crRNAs) (6). Finally, during interference, Cas-crRNA complexes find the complementary sequence in the foreign nucleic acid (the protospacer) and promote its degradation (7). CRISPR–Cas systems are diverse, and are divided into two classes (1 and 2) and six types (I-VI) (8). Class 1 systems form multi-subunit complexes with crRNAs, whereas class 2 utilise a single effector protein. In the current study, we focus on type I and III systems (class 1). Type I systems target DNA, while type III systems degrade both RNA and DNA. The structures and mechanisms of CRISPR–Cas systems, and their biotechnological applications have been studied extensively (9). In contrast, the mechanisms of CRISPR–Cas regulation are less well understood (10,11).

Phage resistance provided by CRISPR–Cas can be beneficial for bacteria; however, self-targeting and inhibition of plasmid uptake can have downsides (12–16). Appropriate regulation of CRISPR–Cas is proposed to be important to maximise defence when required, while limiting potential costs (11). Indeed, a recent study demonstrated that a natural single guide RNA directs Cas9 to autoregulate and tune CRISPR–Cas defence while limiting autoimmunity (17). Bacteria also control CRISPR–Cas through quorum sensing communication to upregulate defence at high cell density when facing a heightened risk of a phage epidemic (18,19). For example, in *Serratia* sp. ATCC 39006 (hereafter *Serratia*), a Gram-negative Enterobacteriaceae, we showed that all three CRISPR–Cas systems (type I-E, I-F and III-A) are controlled by cell-cell communication (18). Although various CRISPR–Cas regulatory pathways have been discovered, this has been limited by the lack of techniques to comprehensively identify regulators of gene expression in bacteria. To overcome this limitation, we recently developed a high-throughput method—termed SorTn-seq—that couples high-density transposon mutagenesis with reporter genes, fluorescence activated cell sorting (FACS), and transposon insertion sequencing to identify regulators of the type III-A CRISPR–Cas system in *Serratia* (20,21). Amongst the multiple genes discovered, transposon insertions within three genes (*pigW*, *pigQ* and *rsmB* (i.e. homologues of *Escherichia coli barA*, *uvrY* and *csrB*, respectively)—belonging to the Rsm (regulator of secondary metabolism) post-transcriptional regulatory pathway—were enriched in cells with low type III-A expression (20).

The Rsm pathway is known as Csr (carbon storage regulator) in *Escherichia coli*, and these regulatory circuits are widespread, particularly in proteobacteria (for reviews see (22–24)). Csr (Rsm) controls multiple processes, such as stress responses, changes in metabolism and carbon utilisation, biofilm formation, virulence and motility (22–24). Similarly, in *Serratia*, mutations in the Rsm pathway affect pigmentation, virulence and motility (inversely controlling gas vesicle-mediated floating versus flagella-mediated swarming) (25–29). CsrA (RsmA in *Serratia*) is the central pathway component and is a dimeric RNA-binding protein that alters translation, RNA stability or transcription elongation (23,24). CsrA binds target mRNAs in GGA motifs, sometimes within a loop structure in, or near, ribosome binding sites (RBS) within the 5′ untranslated region (5′UTR) (23,30). When CsrA binds in this manner, it typically inhibits translation by impeding ribosome loading.

Here, we show that the Rsm pathway regulates all three CRISPR–Cas systems (type I-E, I-F and III-A) in *Serratia*. RsmA binds *cas* mRNAs, potentially altering their stability and/or repressing their translation, thereby reducing CRISPR–Cas interference and adaptation. By coregulating CRISPR–Cas and flagella, the Rsm pathway enables coordinated control of receptor-based versus adaptive immunity. We demonstrate that RsmA-mediated regulation of CRISPR–Cas occurs in other genera, suggesting it could be a more widespread mechanism to control bacterial adaptive immunity. Finally, the identification of RsmA homologues in phages indicates that the Rsm pathway could be exploited by phages to manipulate bacteria, such as to suppress their CRISPR–Cas immune responses.

## MATERIALS AND METHODS

### Strains and growth conditions

The bacterial strains used in this study are listed in Supplementary Table S1. All plasmids are listed in Supplementary Table S2, including details of their construction, and oligonucleotides used are listed in Supplementary Table S3. All strains and plasmids were confirmed by Sanger sequencing. *Serratia* sp. ATCC 39006 strains were grown at 30°C, *Pectobacterium atrosepticum* SCRI1043 at 25°C and *E. coli* strains at 37°C in lysogeny broth (LB) medium and on plates containing 1.5% (w/v) agar (LBA). Media was supplemented with antibiotics at the following concentrations: Kanamycin (Km) at 50 μg/ml; Ampicillin (Ap) at 100 μg/ml; Chloramphenicol (Cm) at 25 μg/ml, Spectinomycin (Sp) at 50 μg/ml, Gentamicin (Gm) at 50 μg/ml and Tetracycline (Tc) at 10 μg/ml. Five-aminolevulinic acid (ALA; 50 μg/ml) was added for growth of ST18. Bacterial growth was measured as optical density using a Jenway 6300 Spectrophotometer at 600 nm (OD_600_). When grown in 96-well plates, plates were incubated in an IncuMix shaker and optical density was measured using a Varioskan LUX Multimode Reader (Thermo Fisher Scientific) at 600 nm.

### Reporter plasmids and gene expression assays

S*erratia* strains, each containing a plasmid with the fluorescent reporter zsGreen fused to a promoter of a different *cas* gene, were used in plate reader assays to measure gene expression (Supplementary Figure S1). The major transcriptional start sites were determined from an in-house unpublished transcriptional start site RNA seq dataset. Plasmids used to measure expression were: *cas3* (type I-E, pPF1973), *cas1* (type I-F, pPF1891), *cas10* (type III-A, pPF1890) and *rsmB* (pPF1976) and were compared with the empty vector (pPF1854). Pigmentless (prodigiosin-deficient) versions of all the strains were used for these assays to remove any overlap with fluorescence measurements (PCF396 Δ*pigA-O* background). Strains were generated by generalised transduction of marked mutations into the PCF396 Δ*pigA-O* background as described previously (31). Mutant derivatives of PCF396 were used in these assays as follows: PCF398 (*rsmA*), PCF406 (*rsmB*), PCF629 (*pigQ*), PCF630 (*pigW*), PCF631 (*pigX*), PCF694 (*rsmS*), PCF703 (*rsmA*, *rsmS*), PCF675 (*rsmA*, *rsmB*), PCF704 (*rsmA*, *pigX*), PCF705 (*rsmS*, *rsmB*), PCF706 (*rsmS*, *pigX*) and PCF717 (*rsmB*, *pigX*). Strains carrying appropriate plasmids were grown in a 96 deep-well plate overnight in 1 ml LB containing Gm. For all reporter assays, the OD_600_ of the overnight cultures was measured and adjusted to 0.02 to inoculate a 96-well black sided, clear bottom plate (with the appropriate antibiotics) to monitor OD_600_ as well as fluorescence with excitation at 490 nm and emission at 510 nm for 23 h. For all endpoint reporter data presented, fluorescence was normalised by the OD_600_.

Complementation assays were performed similarly using the strains listed above, but containing either an empty vector control (pPF781) or the expression vector for each gene: *rsmA* (pPF1958), *rsmB* (pPF1959), *pigQ* (pPF1960), *pigW* (pPF1961), *pigX* (pPF1962) and *rsmS* (pPF1964). Complementation strains were grown in LB containing Gm + Cm.

### Sample and library preparation for RNA sequencing

Overnight cultures of *Serratia* LacA (WT) and the *rsmA* mutant (NMW7) were subcultured into 25 ml LB medium in 250 ml flasks in biological triplicates to a starting OD_600_ of 0.05. The cultures were grown in LB broth for 12 h at 30°C with shaking at 200 rpm, and 2 ml samples were collected for RNA extraction. The twelve-hour time point (early stationary phase) was previously established as a point of elevated CRISPR–Cas activity due to the rising density of bacterial populations (18). Bacterial cells were harvested by centrifugation and the resulting cell pellets were resuspended in RNA*later* (Thermo Fisher Scientific) and stored at -20°C until further processing. The Qiagen RNeasy kit was used to extract total cellular RNA. TurboDNase (Thermo Fisher Scientific) was added to remove genomic DNA (gDNA). Samples were confirmed to be gDNA-free by means of PCR analysis with primers PF796 and PF797 that are designed to amplify the *flhDC* operon of *Serratia*. Quality control checks of the resulting RNA samples were performed using the nanodrop (Thermo Fisher Nanodrop one) and 2100 Bioanalyzer (Agilent Genomics).

Ribosomal RNA (rRNA) was depleted using a RiboCop rRNA depletion kit (Lexogen). Synthesis of antisense cDNA was initiated through ligation of a TruSeq adaptor sequence (Illumina) to the 3′ OH end of the fragmented RNA. Next, the antisense cDNA was purified, followed by a ligation of a 5′ sequencing adaptor to the 3′ end of the antisense cDNA. The cDNA was then amplified using PCR and the resulting products were gel fractionated to satisfy the size requirements for Illumina sequencing. Lastly, cDNA libraries were sequenced with the Illumina HiSeq 2500 V2 Rapid sequencing system to an average depth of around 10 million reads per library, generating an output in the form of 100 bp demultiplexed reads in FASTQ format.

### RNA sequencing data analysis

Generated reads in FASTQ format were initially processed by removing adaptors and low-quality reads using Trimmomatic (32). Additionally, quality assessment of the reads was carried out using FastQC (Babraham Bioinformatics, [https://www.bioinformatics.babraham.ac.uk/projects/fastqc/]). Bowtie2 (33) was used with default parameters for mapping reads to the reference genome of *Serratia* sp. ATCC 39006 (accession number: CP025085), followed by a conversion to BAM format for analysis using SAMtools (34). Statistical analysis was performed using the default settings of the DESeq2 package of R/Bioconductor to identify differentially expressed transcripts with a false discovery rate (FDR) of less than 5% and a fold change >1.5 (i.e. log2 >0.58) (35). Full RNA-seq outputs from DESeq2 are provided in Supplementary Table S4.

### Type III-A CRISPR–Cas interference assays

CRISPR–Cas interference assays are based on conjugation efficiency and were carried out as described previously (18). Briefly, *E. coli* ST18 was used as the donor for conjugation of an untargeted control (pPF781) and type III-A targeting plasmid (pPF1043). Plasmid pPF1043 has a protospacer targeted by a spacer in CRISPR3. Strains were grown overnight in LB with appropriate antibiotics, following which, 1 ml of culture was centrifuged at 17 000 × g and the supernatant removed. Cell pellets were washed with 500 μl LB + ALA to remove antibiotics and resuspended in 1 ml LB + ALA. Washed and resuspended cells were adjusted to an OD_600_ of 1. *E. coli* donors and *Serratia* recipients (WT or NMW7 (*rsmA*)) were mixed in a 1:1 ratio and 20 μl spotted on LBA + ALA + glucose (0.2% w/v) and incubated at 30°C for 20 h. Conjugation spots were scraped from the plate and added to 500 μl phosphate buffered saline (PBS) to resuspend cells. Resuspended cells were serially diluted (undiluted to 10^−8^) and plated onto LBA + arabinose (0.02% w/v) for recipient counts or LBA + arabinose + Cm for selection of transconjugants. Arabinose was included to induce the protospacer transcription necessary for type III-A targeting. CRISPR–Cas interference was assessed using conjugation efficiency, which was measured as a ratio of transconjugants divided by recipients. In addition, to assess the effects of *rsmA* overexpression on interference, conjugation efficiency assays were performed similarly with the following recipients (NMW7 (*rsmA*) + pQE-80L-oriT (control) and NMW7 (*rsmA*) + pPF513 (RsmA)). Bacteria were plated onto LBA + Ap + arabinose (0.02%) for recipient counts or LBA + Ap + Cm + arabinose for selection of transconjugants. Conjugation efficiency data was log-transformed and normality was assessed using Shapiro-Wilk and Kolmogorov-Smirnov tests. A Student's *t*-test (two-sided) was used on log-transformed data to determine statistical significance.

### Type I CRISPR–Cas interference assays

*Serratia* type I conjugation efficiency assays were performed the same as type III, but with the following variations. An untargeted control (pPF719) and type I-E (pPF724) or type I-F (pPF722) targeting plasmids were used. Plasmids pPF724 and pPF722 each contain a protospacer targeted by a spacer from either CRISPR1 (type I-E) or CRISPR2 (type I-F). Recipient *Serratia* strains used were WT (LacA) and an *rsmA* mutant (NMW7). Following conjugation, bacteria were plated onto LBA for recipient counts or LBA + Tc for selection of transconjugants. In addition, to assess the effects of *rsmA* overexpression on interference, conjugation efficiency assays were performed with the following recipients (NMW7 (*rsmA*) + pPF781 (control) and NMW7 (*rsmA*) + pPF1958 (RsmA)), which were grown with arabinose (0.1% w/v). Bacteria were plated onto LBA + Cm + arabinose (0.1% w/v) for recipient counts or LBA + Cm + Tc + arabinose for selection of transconjugants. *Pectobacterium* type I-F conjugation efficiency assays were carried out the same as for *Serratia* type I systems, with the following variations. An untargeted control (pPF571) and type I-F targeting plasmid (pPF572) were used. Plasmid pPF572 contains a protospacer targeted by a spacer from CRISPR1. Strains used were WT (*P. atrosepticum* SCRI1043) and an *rsmA* mutant (AE9). Conjugation efficiency data was log-transformed and normality was assessed using Shapiro-Wilk and Kolmogorov-Smirnov tests. A Student's *t*-test (two-sided) was used on log-transformed data to determine statistical significance.

### Primed CRISPR adaptation assay

Primed CRISPR adaptation was analysed using priming plasmids that lead to acquisition of new spacers. Plasmids (pPF953, naïve; pPF1233, I-E primed; pPF1236, I-F primed) were conjugated into *Serratia* WT (LacA) and *rsmA* (NMW7) from *E. coli* ST18 donors and the transconjugants were grown in LB with Tc selection and 25 μM IPTG for 24 h. The I-E and I-F primed plasmids contain a protospacer with a non-canonical protospacer adjacent motif, that promotes acquisition of new spacers through priming. From those cultures, new overnight cultures without antibiotic selection, but containing IPTG, were inoculated and grown for 24 h. From those cultures, 10 μl were used to inoculate new overnight cultures containing 5 ml of LB without selection and grown for 24 h at 30°C. New cultures were inoculated every 24 h for up to two days to observe priming. Aliquots of culture from each day were mixed 1:1 with 50% glycerol and frozen at -80°C for future use. CRISPR array expansion (indicative of adaptation) was assessed via PCR (20 cycles) with primers PF633/PF2177 for type I-E (CRISPR1) and PF1888/PF1990 for type I-F (CRISPR2). PCR samples were run on a 2% (w/v) agarose gel with ethidium bromide in sodium borate buffer. To assess the effect of *rsmA* expression on CRISPR adaptation, plasmids (pPF953, naïve; pPF1233, I-E primed; pPF1236, I-F primed) were conjugated into strains NMW7 (*rsmA*) + pPF781 (control) and NMW7 (*rsmA*) + pPF1958 (RsmA) in the presence of Cm for expression plasmid maintenance and arabinose (0.1% w/v) for *rsmA* expression. Serial passaging and PCR was performed as described above, with the addition of Cm and arabinose (0.1% w/v) to culture media.

### Construction of a FLAG-tagged RsmA strain

A C-terminal *rsmA-*3xFLAG chromosomal fusion was constructed using allelic exchange mutagenesis with sucrose selection. A plasmid (pPF1811) was constructed using a gBlock (PF3562) containing 500 bp upstream and downstream the *rsmA* gene and the 3xFLAG, and the *sacB*-containing suicide plasmid pPF1117 as a backbone. The plasmid pPF1811 was then conjugated into *Serratia* and plated onto LBA containing Cm to select for recombination of the plasmid with the chromosome. An overnight culture without selection was plated on LBA with 10% (w/v) sucrose to select for recombination leading to plasmid (*sacB*) loss. The *rsmA* region was screened by PCR (PF3563 and PF3565) and the mutant confirmed by Sanger sequencing.

### Co-IP and RIP-seq sample preparation

Co-immunoprecipitation of RsmA-3xFLAG with an anti-FLAG antibody and Protein A-Sepharose beads was performed from *Serratia* lysates of WT (LacA control) and isogenic RsmA-3xFLAG strain (PCF624). The cells were grown overnight and 100 ml LB cultures were inoculated to an OD_600_ of 0.05 in 500 ml flasks and grown to late exponential phase (OD_600_ of 0.6) at 30°C as described earlier. Cells were harvested by centrifugation at 6000 × g for 20 min at 4°C. The cell pellets corresponding to a total OD_600_ of 60 were then resuspended in 1 ml of Buffer A (20 mM Tris–HCl pH 8.0, 150 mM KCl, 1 mM MgCl_2_ and 1 mM dithiothreitol (DTT)) and subsequently harvested by centrifugation (10 min, 11 000 × g, 4°C). The pellets were snap-frozen in liquid nitrogen and stored at -80°C.

When required, the pellets were thawed on ice and resuspended in 1 ml of lysis buffer (Buffer A containing 1 mM PMSF, 0.2% (v/v) Triton X-100, DNase I 0.02 U/μl and RNase inhibitor 0.4 U/μl) and transferred to FastPrep tubes (MP biomedicals) containing silica spheres. Cells were lysed using FastPrep24 running two lysis steps at the speed of 4 m/s for 15 s, with 5 min cooling on ice between each step. Cell debris was then separated by centrifugation at 17 000 × g for 10 min at 4°C and the supernatant (lysate fraction) was collected into a new tube. Incubation of the samples with 35 μl of anti-FLAG antibody (Monoclonal anti-FLAG M2, Sigma, #F1804) was carried out for 30 min at 4°C on an orbital shaker. Then 75 μl of ProteinA-Sepharose (Sigma P6649 6MB aqueous ethanol suspension) were washed with Buffer A and added to the lysate containing the antibody. Solutions were then incubated for a further 30 min at 4°C on an orbital shaker. The beads with the lysate were then subjected to centrifugation at 15 000 × g for 1 min at 4°C. After centrifugation, the supernatant was removed. The beads were washed 5 times with 500 μl of Buffer A. A phenol–chloroform–isoamyl (PCI) extraction was performed, adding 500 μl of Buffer A and 500 μl of PCI to the beads, followed by inversion of the tube and 5 min incubation at room temperature (RT). A 30 min centrifugation step at 17 000 × g at 4°C was performed to pellet the RNA.

An overnight precipitation at –20°C was performed by adding the supernatant to a tube containing 1 ml of 30:1 mix (absolute ethanol:sodium acetate) plus 1 μl of GlycoBlue. The RNA precipitate was subjected to centrifugation at 17 000 × g for 30 min at 4°C and washed with 500 μl of ice-cold 70% ethanol followed by a further 10 min centrifugation in the same conditions. Then the pellet was left to dry by opening the tube at room temperature. A DNase I digestion was carried out to eliminate all possible DNA, followed by a PCI extraction, overnight precipitation in 30:1 mix (absolute ethanol:sodium acetate) and final wash in 70% ice-cold ethanol as previously explained. The absence of DNA contamination was confirmed by PCR of the *pigQ* gene (PF3143 and PF3144).

### SDS-PAGE and western blotting of RsmA-3xFLAG

Protein samples (equivalent to an OD_600_ of 1) were collected during Co-IP experiments to confirm enrichment of RsmA-3xFLAG by western blotting. These cell pellets were resuspended in the appropriate volume of protein loading buffer (62.5 mM Tris–HCl pH 6.8, 100 mM DTT, 10% (v/v) glycerol, 2% (w/v) SDS and 0.01% (w/v) bromophenol blue) to make final concentration of 0.1 OD_600_/μl per sample and boiled at 95°C for 8 min. A total volume of 20 μl for culture protein (C), lysate (L), supernatant 1 (SN1), supernatant 2 (SN2) and wash (W) fractions (corresponding to 0.2 OD_600_) and 20 μl from the elution (E) fraction (equivalent to an OD_600_ of 10) were resolved using 15% SDS-polyacrylamide gels and transferred onto nitrocellulose membrane using semi-dry blotting. After transfer, membranes were blocked for 1 h in a 10% milk powder solution in 1× phosphate-buffered saline containing 0.01% sodium azide (PBS-A) buffer followed by washing. An overnight incubation at 4°C of the primary antibody (monoclonal anti-FLAG 1:1000 or monoclonal antibody specific for GroEL 1:10 000 were diluted in PBS-A) was performed, followed by washing with 1× Tris-buffered saline with 0.001% Tween 20 (TBST20) and incubation with the horseradish peroxidase (HRP)-coupled secondary antibody (anti-mouse IgG 1:10 000 in TBST20 for anti-FLAG or anti-rabbit IgG 1:10 000 in TBST20 for anti-GroEL) for 1 h at 4°C. After washing, the membrane was developed using the HRP catalyzed oxidation of its substrate luminol, in presence of hydrogen peroxidase and *p*-hydroxicoumaric acid as an enhancer. The chemiluminescence output was detected using a CCD camera-equipped Image Quant LAS 4000 machine.

### RIP-seq and data analysis

RNA was converted to cDNA using the adapter ligation method by Vertis Biotechnologie AG, Germany (http://www.vertis-biotech.com). Briefly, the eluted RNA after DNase I treatment was subjected to oligonucleotide adapter ligation on the 3′ end, first-strand cDNA synthesis using M-MLV reverse transcriptase (Agilent), and Illumina TruSeq sequencing adapter ligation on the 3′ end. The resulting cDNA was PCR-amplified using Herculase II Fusion DNA Polymerase (Agilent) with 13 amplification cycles following the manufacturer's instructions, purified using Agencourt AMPure XP kit (Beckman Coulter Genomics) following the manufacturer's instructions, and analyzed by capillary electrophoresis. After cDNA purification and quantification with capillary electrophoresis, two cDNA pools were constructed, according to ratios calculated from the quantification, for Illumina NextSeq sequencing (data collected from a protocol provided by Vertis Biotechnologie AG). The resulting samples were then run on an Illumina NextSeq 500 instrument with 76 cycles in single-read mode (Supplementary Table S5).

RIP-seq data analysis was performed as follows. First, demultiplexing was performed using bcl2fastq v2.20.0.422. Illumina reads were quality controlled and adapter trimmed with Cutadapt (36) version 2.5 using a cutoff Phred score of 20 in NextSeq mode and reads without any remaining bases were discarded (command line parameters: --nextseq-trim=20 -m 1 -a AGATCGGAAGAGCACACGTCTGAACTCCAGTCAC). Afterwards, we applied the pipeline READemption (37) version 0.4.5 to align all reads longer than 11 nt (-l 12) to the *Serratia* reference genome (RefSeq assembly accession: GCF_002847015.1) using segemehl version 0.2.0 (38) with an accuracy cut-off of 95% (-a 95). We used READemption gene_quanti to quantify aligned reads overlapping genomic features by at least 10 nts (-o 10) on the sense strand (-a). For this, we applied RefSeq annotations (antisense_RNA, CDS, ncRNA, riboswitch, RNase_P_RNA, rRNA, SRP_RNA, tmRNA, tRNA, direct_repeat) for assembly GCF_002847015.1 (annotation date 16 March 2017) in GFF format supplemented with annotations for strand-specific intergenic regions, and sRNAs based upon homology to *Salmonella* sRNAs. For annotation-based identification of enriched genomic regions based on biological duplicates, we used DESeq2 (35) version 1.24.0. Size factors for normalization were calculated manually by dividing the total number of aligned reads for each library by the minimum over all libraries. Fold-change shrinkage was applied by setting ‘betaPrior = TRUE’ and testing only for enrichment in the RsmA-3xFLAG compared to the WT CoIP libraries was conducted by setting altHypothesis = ‘greater’. Features were considered significantly enriched with a adjusted *P*-value <0.005 (Supplementary Table S6). In addition, we applied READemption to generate coverage plots representing the numbers of mapped reads per nucleotide. Here, we used sequencing depth-normalized plots from output folder coverage-tnoar_min_normalized for visualization in the Integrated Genome Browser (IGB) (39).

### Peak calling and RsmA motif analysis

Peak calling for RIP-seq libraries was performed using the sliding window approach implemented in the tool PEAKachu (https://github.com/tbischler/PEAKachu, version 0.1.0, commit c869dc5583c0ccd9981c0576d38ce388f2df958c). The tool was run using as input READemption BAM files for the two replicates of RsmA-3xFLAG and WT, respectively. By calling *peakachu window*, we calculated library-specific read count values of genomic regions using window size (-w) 25 and step size (-l) 5. Window count normalization and detection of significantly enriched windows subsequently merged into peaks was conducted via methods implemented in DESeq2 (35) using the following PEAKachu parameters: normalization method (-n) manual, statistical test (-d) deseq, mad-multiplier (-m) 6.0, fold change (-f) 10.0 and Benjamini-Hochberg adjusted *P*-value (-Q) 0.05. Size factors for normalization (-s) were calculated by dividing the total number of aligned reads for each library by the minimum over all libraries and annotations in GFF format (see above) were used to map overlapping features to called peaks. Enriched Co-IP sequences from PEAKachu that reached significance were analyzed by MEME version 5.2.0 to generate consensus motifs (http://meme-suite.org/) (40). The analysis was run using default settings, except for restricting the motif width to 6-10 characters.

### Phage infection assays

To test for the effects of RsmA on phage sensitivity independently of CRISPR immunity, the WT (LacA) and an *rsmA* mutant (NMW7) were grown overnight in LB with no supplements. Stationary phase cultures were normalised to an OD_600_ of 0.05 in 180 μl LB in a 96-well plate. Dilutions of ΦJS26 lysate were prepared in phage buffer (10 mM Tris–HCl pH 7.4, 10 mM MgSO_4_ and 0.01% (w/v) gelatine) and 20 μl was added to each well to produce the desired MOI. For the uninfected controls, 20 μl of phage buffer was added to wells. Plates (96-well) were incubated in a plate reader (Varioskan Flash, Thermo Scientific) at 30°C with shaking at 300 rpm and OD_600_ measurements taken every 12 min for 20 h. Each condition was repeated in triplicate with data plotted as the mean +/− the standard deviation.

To assess CRISPR–Cas protection against phage infection, plasmids harbouring mini-CRISPR arrays expressing anti-ΦJS26 spacers (pPF1473 - type III; pPF1485 - type I-E; pPF1489 - type I-F) or a control plasmid containing no mini-CRISPR array (pPF260 - control) were introduced into strains WT (LacA) and an *rsmA* mutant (NW64). Strains were grown overnight in LB with kanamycin (50 μg/ml) for plasmid maintenance and IPTG (0.1 mM) for CRISPR-array expression. Stationary phase cultures were normalised to an OD_600_ of 0.05 in 180 μl LB + kanamycin + IPTG in a 96-well plate and the growth conditions performed as described in the previous paragraph.

### Identification and analyses of RsmA in phage genomes

We first searched Caudovirale, Kalamavirale, Levivirale, Mindivirale, Tubulavirale and Vinavirale genomes in GenBank for RsmA homologs via a hidden Markov model (HMM) search using the Pfam CsrA/RsmA HMM (PF02599) with HMMER3 (hmmer.org). We used a full sequence *E*-value cut-off of 10^−4^ and a 50% coverage threshold for the HMM and 30% coverage of the target sequence. The phage CsrA/RsmA sequences were filtered to remove duplicates using CD-HIT (41), aligned using MUSCLE (42), manually curated, then used to build a phage-CsrA/RsmA HMM with HMMER3. The phage-CsrA/RsmA HMM was used to search the GenBank Caudovirale, Kalamavirale, Levivirale, Mindivirale, Tubulavirale and Vinavirale genomes and non-eukaryotic viral contigs in the IMG/VR database v3 (September 2020). We applied a full sequence *E*-value cut-off of 10^−4^ and a 50% coverage threshold for the HMM and 30% coverage of the target sequence. Classification of whether host bacteria encoded CsrA/RsmA was based on the prevalence of RsmA homologs within sequenced genomes (RefSeq) of the corresponding host taxonomic genus. First, we used an HMM search (as above) to determine whether each bacterial genome in RefSeq (September 2020) encoded CsrA/RsmA. For each bacterial genus, based on the NCBI taxonomy, we then determined the proportion of CsrA/RsmA-encoding genomes, which revealed that CsrA/RsmA is generally either very common or almost entirely absent from any given genus (Supplementary Figure S9). Genera where <20% of genomes encoded CsrA/RsmA were classed as typically lacking CsrA/RsmA, whereas genera with >80% of genomes encoding CsrA/RsmA were classed as hosts with CsrA/RsmA. Alignment of CsrA/RsmA homologs was performed using MUSCLE (42). Protein accessions (NCBI) for Figure 6E are: NP_417176.1 (*E. coli* K12), WP_005972168.1 (*Serratia* ATCC 39006), QBQ72211.1 (*Serratia* phage Parlo), AXF51437.1 (*Erwinia* phage Pavtok), AEZ50864.1 (*Burkholderia* phage DC1), AXG67699.1 (*Ralstonia* phage GP4) and QIW86647.1 (*Klebsiella* phage LASTA).

## RESULTS

### The Rsm pathway regulates the type III-A CRISPR–Cas system

**Figure 1.**
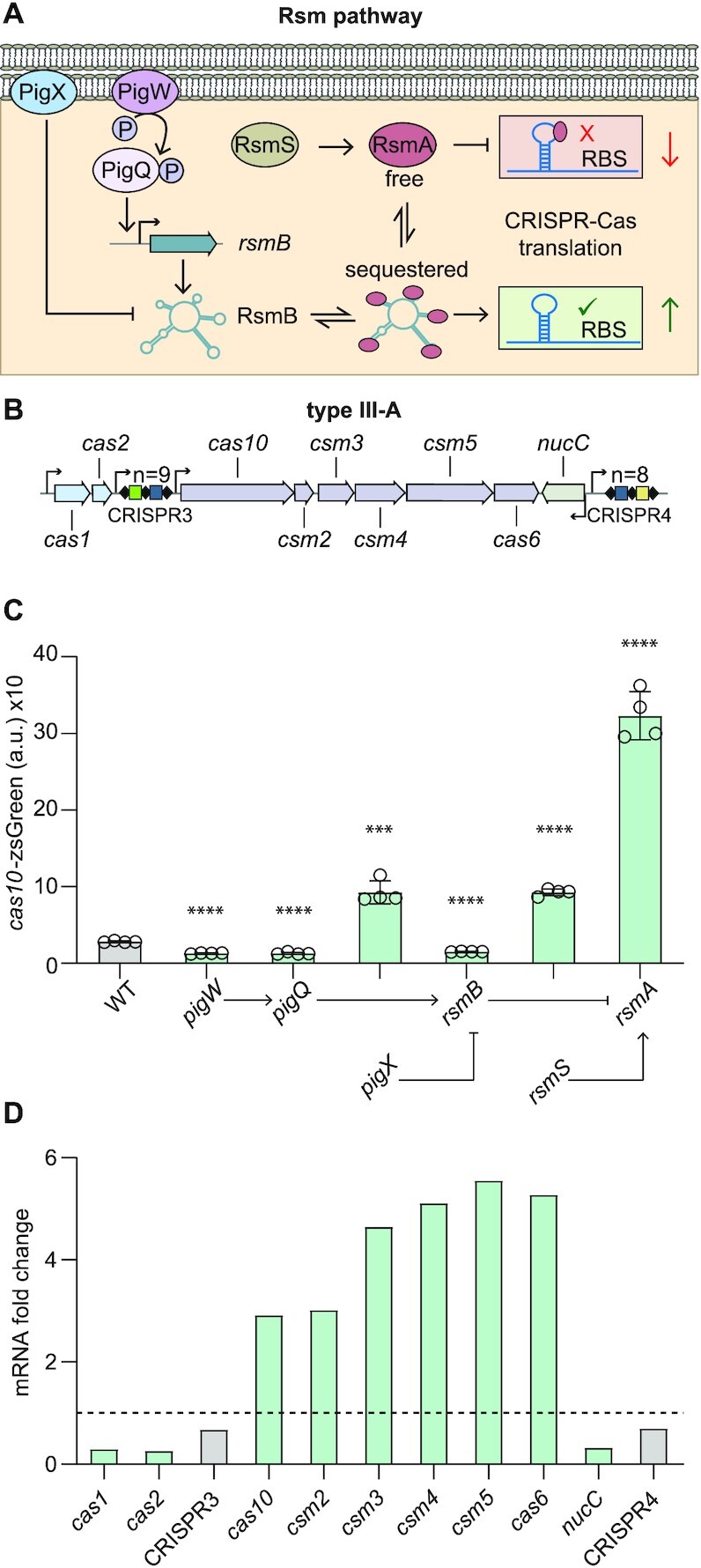
The Rsm (Csr) pathway regulates type III CRISPR–Cas. (**A**) Overview of the Rsm pathway. Gene names provided in the figure and legend are for *Serratia*. In the following description the *E. coli* homologues are in parentheses. The histidine kinase, PigW (BarA), phosphorylates the response regulator, PigQ (UvrY). PigQ∼P activates transcription of the non-coding RNA, RsmB (CsrB/C). RsmB sequesters and inhibits the RNA binding protein, RsmA (CsrA). RsmB can be degraded via PigX (CsrD) (and RNaseE). The activity of RsmA is also supported by RsmS (YbaM) through direct protein interactions. RsmA typically binds mRNA to inhibit translation, but can have other effects on expression. (**B**) Schematic of the *Serratia* type III-A CRISPR–Cas system. Arrows represent the promoters for the *cas* genes and CRISPR arrays. n = number of spacers. (**C**) Mutation of genes encoding the Rsm components affects *cas10* (*csm*) expression (a.u., arbitrary units; *n* = 4 biologically independent samples). Pathway organisation is indicated below the graph. All bars represent the mean and error bars represent the standard deviation. To determine statistical significance, two-sided *t*-tests were used to compare the mutants with the WT (****P < 0.0001; ***P < 0.001; **P < 0.01). See also Supplementary Figures S1, S2. (**D**) RNA fold changes determined by RNA-seq in the type III-A CRISPR–Cas region for the *rsmA* mutant compared with the WT. Dashed line represents no change relative to WT (i.e. fold change = 1). Grey bars indicate features outside of the significance and fold change threshold (see Materials and Methods). For full RNA-seq (DESeq2) data and statistics see Supplementary Table S4.

In our SorTn-seq screen to find regulators of the *Serratia* type III-A CRISPR–Cas system, we identified transposon insertions in Rsm pathway genes *pigW* (*barA*), *pigQ* (*uvrY*) and *rsmB* (*csrB*) (20). The activity of CsrA (RsmA) proteins is controlled by inhibitory non-coding RNAs (ncRNAs) that bind and sequester multiple CsrA dimers to out-compete mRNA targets (30) (Figure 1A). *E. coli* has two such ncRNAs, CsrB and CsrC, and their transcription is regulated by the BarA/UvrY GacAS-family two-component signal transduction system (43). In *Serratia*, the homologous two-component system is PigW/PigQ and RsmB is a CsrB homologue (Figure 1A). As these genes are part of the Rsm pathway (25,27), we proposed that this regulatory cascade affected type III-A CRISPR–Cas expression (Figure 1B). Although RsmA was not identified in the SorTn-Seq screen (due to small gene size and reduced fitness in mixed culture), it is the central player in the Rsm pathway, combining signals from multiple inputs. Since RsmA acts post-transcriptionally (23), a transcriptional/translational reporter was constructed by fusing the major promoter (*cas10*) and the 5′ UTR including the native RBS of the type III-A *cas* (aka *csm*) interference operon to a fluorescent reporter (zsGreen) (Supplementary Figure S1A, B).

Expression of the *cas10* reporter was elevated ∼10-fold in an *rsmA* (*csrA*) mutant compared with the wild-type (WT), demonstrating that RsmA represses the type III-A *cas* operon (Figure 1C, Supplementary Figure S2A). Based on work in *E. coli*, we predicted and then demonstrated that the *Serratia* PigQW two-component system activates expression of RsmB (Supplementary Figure S2B). This is consistent with our identification of a putative PigQ inverted repeat binding site upstream of the *rsmB* promoter (TGTAAGATATCTCTTACA), which is an 18/20 nt match with the UvrY-box in *E. coli* (43). In agreement, mutation of *rsmB* or the genes encoding the PigQW two-component system resulted in lower *cas* expression (Figure 1C, Supplementary Figure S2A) – i.e. reduced levels of the RsmB ncRNA results in increased free RsmA, which leads to stronger *cas* repression.

In *E. coli*, the membrane-bound CsrD protein controls CsrB/C post-transcriptionally by interacting with the sugar phosphotransferase (PTS) system EIIA^Glc^ during glucose uptake and stimulates RNaseE-mediated decay of CsrB/C (44,45). In a *Serratia pigX* (*csrD*) mutant, RsmB was elevated and *cas* expression increased, likely due to sequestration of RsmA (Figure 1C, Supplementary Figure S2A, B). These results are consistent with increased RsmB RNA in *pigX* (and *csrD*) mutants (27,44). Another protein, RsmS (*E. coli* YbaM), was recently shown to directly interact with RsmA in *Pectobacterium*, and potentially increases the efficiency of RsmA binding to target mRNAs (46). In agreement, mutation of *rsmS* in *Serratia* resulted in higher type III-A *cas* expression, likely due to decreased RsmA activity (46) (Figure 1C, Supplementary Figure S2A).

RNA binding by CsrA (RsmA) can influence not only translation, but also RNA stability (23,24). Therefore, we examined the effects of the Rsm pathway on mRNA abundance for each gene within the type III-A CRISPR–Cas system by RNA sequencing (RNA-seq) the *rsmA* mutant compared with the WT in early stationary phase (Supplementary Table S4). Transcript levels for each gene within the interference operon that is expressed from the *cas10* promoter (*cas10*, *csm2*, *csm3*, *csm4*, *csm5* and *cas6*) were significantly elevated by approximately 3- to 5.5-fold in the *rsmA* mutant (Figure 1D). Therefore, the effects of the Rsm pathway on the *cas10* transcriptional/translational reporter correlate with an accumulation of *csm* interference gene transcripts. This type III system has a proposed accessory nuclease (*nucC*) convergently transcribed at the end of the *csm* operon (47), which, in contrast, showed a decrease in mRNA abundance (Figure 1D). Similarly, *cas1* and *cas2*, which are expected to be involved in type III CRISPR adaptation and are expressed from a very weak promoter (18) displayed lower mRNA levels in the *rsmA* mutant (Figure 1D). Next, we examined the effects of RsmA on the type III CRISPR arrays (CRISPR3/CRISPR4). Both CRISPR arrays showed no significant change in RNA levels in the *rsmA* mutant when compared with the WT (Figure 1D). In conclusion, the Rsm pathway controls the type III-A CRISPR–Cas system in *Serratia* by repressing interference *cas* gene expression and affecting mRNA abundance.

### Modulation of free RsmA levels regulates type III-A CRISPR–Cas

The Rsm (Csr) pathway is well characterised in *E. coli* but is less understood in *Serratia*. To verify the role of the entire Rsm pathway in type III expression, we first complemented each mutant with the corresponding gene expressed from a plasmid and assessed *cas10*-zsGreen expression (Supplementary Figure S3). All genes functioned as expected, either restoring *cas* expression in the mutants to WT levels, or further decreasing or increasing expression, depending on their positive or negative role in the pathway (Supplementary Figure S3). Our data suggested that the organisation of the *Serratia* Rsm pathway was similar to *E. coli*. Indeed, *Serratia* RsmA shares 98% amino acid identity with *E. coli* CsrA. Therefore, the different genes in the pathway were expected to exert their effects by controlling the availability of active RsmA. Indeed, we observed *cas* repression with increased RsmA expression (Supplementary Figure S3). In contrast, expression of RsmB caused increased *cas* expression, predicted to be due to RsmA sequestration (Supplementary Figure S3). In contrast to the two anti-CsrA ncRNAs in *E. coli*, *Serratia* appears to have a single antagonistic ncRNA (RsmB), since mutations in *rsmB*, *pigQ* or *pigW* reduce *cas* expression to similar levels (Figure 1C).

**Figure 2.**
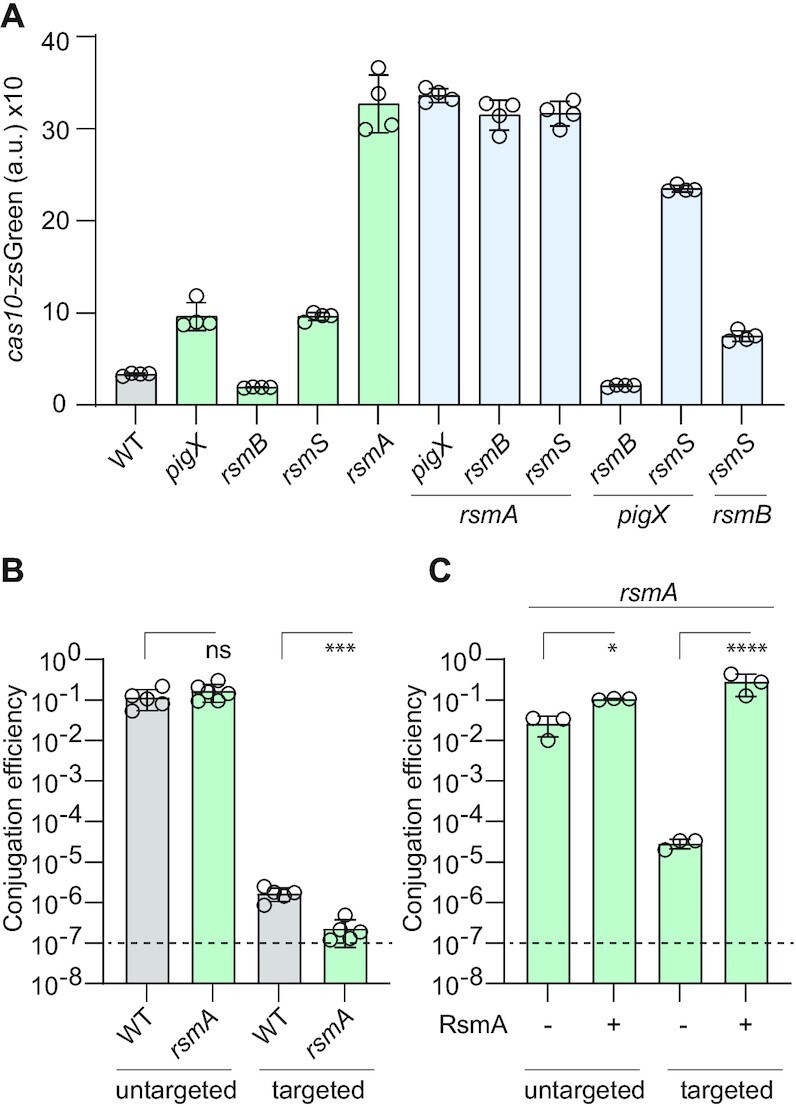
The Rsm pathway acts through RsmA to regulate type III CRISPR–Cas interference. (**A**) Mutation of *rsmA* is epistatic to other genes in the Rsm pathway for type III *cas* expression. Expression of *cas10* (*csm*) was assessed in single *pigX*, *rsmB* and *rsmS* mutants for the three different branches of the pathway (see Figure 1A), the *rsmA* mutant and in various double mutant combinations (a.u., arbitrary units; *n* = 4 biologically independent samples). (**B**) RsmA represses type III-A CRISPR–Cas interference. CRISPR–Cas interference in the WT and an *rsmA* mutant measured as conjugation efficiency of a plasmid with a protospacer targeted by the CRISPR3 array (targeted) or a control lacking a protospacer (untargeted) (*n* = 5 biologically independent samples). (**C**) CRISPR–Cas interference measured by conjugation efficiency of a plasmid targeted by the CRISPR3 array (targeted) or a control lacking a protospacer (untargeted) in the absence (–, *rsmA* mutant background with vector control) or presence (+, *rsmA* mutant with a plasmid encoding RsmA) of RsmA (*n* = 3 biologically independent samples). All bars represent the mean and error bars represent the standard deviation. To determine statistical significance, two-sided *t*-tests were used for (A) and on the log-transformed data for (B) and (C) (****P < 0.0001; ***P < 0.001; *P < 0.05; ns = not significant). For clarity, *P* values for the relevant statistical tests for (A) are provided in the main text. In (B) and (C) the dashed lines indicate the limit of detection.

To further test the role of each component in the pathway, double mutants were analysed for *cas* expression. We confirmed that RsmA is the dominant member of the *Serratia* Rsm pathway, because the same phenotype was observed for the *rsmA* single mutant or in combination with all other key mutations (i.e. no difference in two sided *t*-tests) (Figure 2A). PigX (CsrD) decreases RsmB levels (27,44) (Supplementary Figure S2B), and in agreement, the elevated *cas* expression in the *pigX* mutant is lost upon *rsmB* mutation (two sided *t*-test; *P* < 0.0001) (Figure 2A). RsmS is the least characterised Rsm member, but it binds RsmA and assists its function (46). Mutation of *rsmB* in the *rsmS* background caused a slight reduction in *cas* expression compared with an *rsmS* single mutant (two sided *t*-test; *P* < 0.001), supporting that RsmS influences RsmA independently of RsmB (Figure 2A). Confirming that PigX and RsmS act independently on RsmA to affect *cas* expression, a *pigX*, *rsmS* double mutant showed elevated expression compared to the single mutants (two sided *t*-test; *P* < 0.0001) (Figure 2A). We reason that this double mutant has low levels of free active RsmA (due to abundant RsmB in the absence of PigX-mediated degradation) and since the absence of RsmS will reduce RsmA function (46). Together, these results demonstrate that members of the *Serratia* Rsm pathway affect type III-A *cas* expression by modulating the activity or availability of free RsmA.

### RsmA represses type III-A CRISPR–Cas interference

Since the Rsm pathway affects type III-A *cas* genes via RsmA, we hypothesised that CRISPR interference would be affected by RsmA. To elicit interference, type III complexes first bind RNA complementary to the crRNA, triggering non-specific cleavage of nearby ssDNA (via the Cas10 HD domain) (48–50). Upon binding to target RNA, the Cas10 Palm domain produces cyclic oligoadenylate signals that activate separate accessory non-specific RNases or DNases (51–57). The non-specific nucleic acid degradation is thought to induce dormancy and ‘buy time’ for Cas10 to clear invader DNA (58,59). This response is self-limiting, being shut off when invader DNA and RNA are destroyed.

To test if RsmA-mediated control of CRISPR–Cas expression affects interference, we measured the ability of the type III-A system to interfere with plasmid uptake via conjugation. Conjugation efficiency of a targeted plasmid containing a transcribed protospacer that is complementary to the crRNA from spacer 1 in the native CRISPR3 array was compared with an untargeted control (Figure 2B). As expected, conjugation of the untargeted plasmid was similar between the WT and *rsmA* mutant (Figure 2B), indicating that mutation of *rsmA* has no significant CRISPR-independent effects on plasmid acquisition. The WT displayed strong type III-A CRISPR–Cas interference when the plasmid had the matching protospacer target (Figure 2B). As predicted, mutation of *rsmA* significantly increased interference, and led to an ∼10-fold reduction in conjugation efficiency (Figure 2B). Since interference is highly efficient in this assay and is reaching the limit of detection, it is difficult to see any further increase in interference. Similarly, under conditions where RsmA is sequestered by RsmB, mutation of *rsmA* may only display a subtle effect. In contrast, expression of RsmA *in trans* should overcome sequestration by RsmB and result in increased RsmA-mediated suppression of type III-A CRISPR–Cas interference. Indeed, expression of RsmA in the *rsmA* mutant completely abolished interference (i.e. a ∼10^4^-fold decrease), confirming the role of RsmA in inhibiting type III-A CRISPR interference (Figure 2C). There was a minor increase in uptake of the untargeted control plasmid upon RsmA overexpression, potentially due some CRISPR independent *rsmA* effects (Figure 2C). Previously, in *Serratia* we demonstrated that the Palm (cOA) and NucC activities are the major factors to elicit plasmid immunity, whereas the HD domain only has a minimal role (47). Overall, our current data demonstrate that the elevated type III interference gene expression in the *rsmA* mutant leads to increased interference, despite a decrease in the mRNA encoding the NucC accessory nuclease (Figure 1D). In summary, RsmA represses the interference activities of the type III-A CRISPR–Cas system in *Serratia*, leading to increased acquisition of plasmids by conjugation.

### The Rsm pathway regulates the type I CRISPR–Cas systems

**Figure 3.**
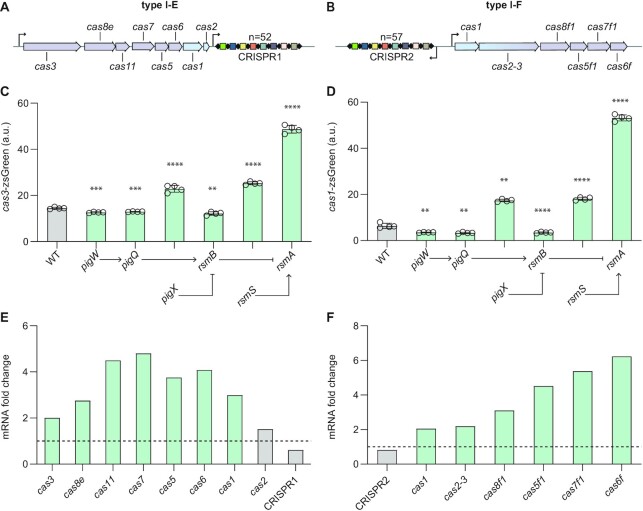
The Rsm pathway represses both type I CRISPR–Cas systems. Schematic of the (**A**) type I-E and (**B**) type I-F *Serratia* CRISPR–Cas systems, with the *cas* and CRISPR promoters indicated by arrows. *n* = number of spacers per CRISPR array. (**C**) Expression of a type I-E *cas3* promoter-zsGreen fusion in Rsm pathway mutants (*n* = 4 biologically independent samples). (**D**) Expression of a type I-F *cas1* promoter-zsGreen fusion in Rsm pathway mutants and the WT (*n* = 4 biologically independent samples). a.u., arbitrary units. All bars represent the mean and error bars represent the standard deviation. To determine statistical significance, two-sided *t*-tests were used to compare the mutants with the WT (****P < 0.0001; ***P < 0.001; **P < 0.01). See also Supplementary Figure S4. RNA fold changes determined by RNA-seq in the (**E**) type I-E and (**F**) type I-F CRISPR–Cas regions for the *rsmA* mutant compared with the WT. Dashed line represents no change relative to WT (i.e. fold change = 1). Grey bars indicate features outside of the significance and fold change threshold (see Materials and Methods). For full RNA-seq (DESeq2) data and statistics see Supplementary Table S4.

In addition to the type III-A CRISPR–Cas system, *Serratia* also encodes DNA-targeting type I-E and I-F systems (Figure 3A, B). Previously, we observed coordinate regulation of all three CRISPR–Cas systems by quorum sensing (18) and the Rcs membrane stress response (20), and we hypothesised that the Rsm pathway may act similarly. To test for Rsm-mediated control of type I CRISPR–Cas immunity, we generated reporter plasmids of the type I-E and I-F operons (Supplementary Figure S1A, C, D). The major promoter of the type I-F operon (*cas1*) including its 5′ UTR and native RBS was fused to zsGreen to create a transcriptional and translational reporter (Supplementary Figure S1C). To detect expression from the major promoter of the type I-E operon (*cas3*) it was necessary to omit 79 nt of the 5′ UTR containing the native RBS and replace it with an artificial RBS (Supplementary Figure S1D). Similar trends in type I *cas* gene expression were detected in the different Rsm pathway mutants as observed for type III-A expression (Figure 3C, D, Supplementary Figure S4A, B). Specifically, mutation of *rsmA, pigX* or *rsmS* led to significantly increased *cas* expression, whereas *rsmB*, *pigQ* or *pigW* mutants displayed significantly lower *cas* reporter activity.

To examine if the Rsm pathway influenced mRNA abundance for each *cas* gene within the type I CRISPR–Cas systems, we analysed the transcriptome of the *rsmA* mutant (Supplementary Table S4). For the type I-E system, mRNA levels of all *cas* genes except *cas2* were significantly higher (ranging from ∼2- to 5-fold) in the *rsmA* mutant compared with the WT (Figure 3E). Similar effects were observed in the type I-F system, with the mRNA abundance of all *cas* genes significantly higher in the absence of RsmA (∼2- to 6-fold) (Figure 3F). These results support the type I *cas* reporter data in the *rsmA* mutant and further demonstrate the accumulation of transcripts from all genes within both type I systems. The effect of RsmA is independent of the CRISPR arrays since RNA levels of both CRISPR1 (type I-E) and CRISPR2 (type I-F) were not significantly altered in the *rsmA* mutant (Figure 3E, F). Therefore, the Rsm pathway regulates both type I and the type III CRISPR–Cas defence systems in *Serratia*.

### RsmA represses type I CRISPR–Cas interference and adaptation

**Figure 4.**
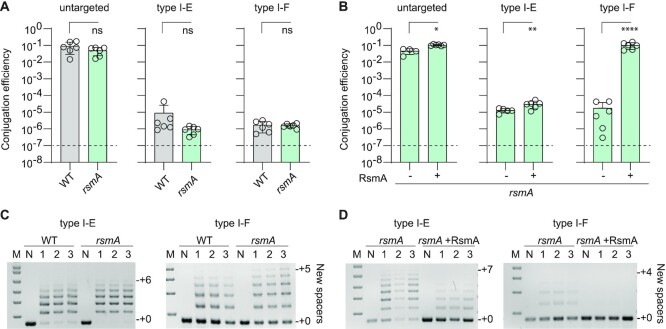
RsmA represses type I CRISPR–Cas interference and adaptation. (**A**) Type I-E and I-F CRISPR–Cas interference in the WT and an *rsmA* mutant measured as conjugation efficiency of plasmids with a protospacer targeted by the CRISPR1 (I-E) and CRISPR2 (I-F) arrays (targeted) or a control lacking a protospacer (untargeted) (*n* = 6 biologically independent samples). (**B**) Type I CRISPR–Cas interference measured by conjugation efficiency of plasmids with a protospacer targeted by the CRISPR1 (I-E) and CRISPR2 (I-F) arrays (targeted) or control (untargeted) in the absence (−, *rsmA* mutant background with vector control) or presence (+, *rsmA* mutant with a plasmid encoding RsmA) of RsmA (*n* = 6 biologically independent samples). In (A) and (B), all bars represent the mean and error bars represent the standard deviation and the dashed lines indicate the limit of detection. To determine statistical significance, two-sided *t*-tests were used on log-transformed data (****P < 0.0001; **P < 0.01; *P < 0.05; ns = not significant). (**C**) CRISPR adaptation in the WT and *rsmA* mutant measured by expansion of CRISPR1 (I-E; left) and CRISPR2 (I-F; right) arrays following one (I-E) or two (I-F) days of growth with a priming-inducing plasmid or naive control plasmid (*n* = 3 biologically independent samples in lanes 1, 2 and 3). (**D**) CRISPR adaptation measured by expansion of CRISPR1 (I-E; left) and CRISPR2 (I-F; right) arrays following one day of growth with a priming-inducing plasmid or naive control in the absence (–, *rsmA* mutant background with vector control) or presence (+, *rsmA* mutant with a plasmid encoding RsmA) of RsmA (*n* = 3 biologically independent samples in lanes 1, 2 and 3). In (C) and (D), N = naive control; M = molecular weight marker.

Since the Rsm pathway regulated *cas* genes involved in interference of both type I systems, we predicted that RsmA would control type I interference capabilities. Interference was tested by measuring conjugation efficiency of plasmids targeted by crRNAs derived from spacer 1 in either CRISPR array of the type I-E or I-F systems (CRISPR1 and CRISPR2, respectively) in comparison to an untargeted control. As predicted, the untargeted plasmid conjugated efficiently into both WT and the *rsmA* mutant (Figure 4A). In contrast, the WT had robust type I-E and I-F interference against plasmids containing a matching target sequence (Figure 4A). We observed no statistically significant increase in type I-E or I-F interference in the *rsmA* mutant (Figure 4A). However, the robust WT interference is already nearing the limits of detection in this assay, making it difficult to identify further increases in interference (Figure 4A; dashed line). Therefore, as with the type III system, we overexpressed RsmA *in trans* to clarify its role in CRISPR–Cas interference. Conjugation efficiency assays were performed in the presence or absence of RsmA expressed from a plasmid in an *rsmA* mutant background. The untargeted plasmid conjugated efficiently irrespective of whether RsmA was present (Figure 4B). Although there was a small, yet significant, decrease in type I-E CRISPR interference in the presence of RsmA, a similar reduction was present in the untargeted control (Figure 4B). Therefore, the regulatory effects of the Rsm pathway on type I-E CRISPR–Cas did not correlate with plasmid interference under these conditions. In contrast, and similar to the type III-A system, RsmA expression in the *rsmA* mutant led to complete repression of type I-F interference (i.e. a ∼10^4^-fold decrease) (Figure 4B). Therefore, RsmA represses interference of type I-F system, but effects on type I-E interference were not detectable under these conditions.

Since the altered type I *cas* levels in Rsm pathway mutants includes proteins involved in spacer acquisition (Cas1 and Cas2 for I-E and Cas1 and Cas2-3 for I-F), we hypothesised that CRISPR adaptation would be affected by RsmA. To measure CRISPR adaptation, a priming assay was performed by introducing plasmids that stimulate new spacer acquisition due to imperfect targeting (60,61). The effects of RsmA on CRISPR adaptation were examined using a priming plasmid for each system (type I-E and I-F) as well as a naïve (untargeted) control plasmid with no matching protospacer. Acquisition of new spacers was visualised as CRISPR array expansion in the bacterial population for the type I arrays (CRISPR1 and CRISPR2) using PCR (Figure 4C). Compared with the WT, the *rsmA* mutant shows a small increase in priming, with more CRISPR array expansion for both type I-E and I-F systems (Figure 4C). Because the RsmA phenotypes were easier to detect with RsmA expressed *in* trans, we performed similar priming assays with or without RsmA expression in the *rsmA* mutant (Figure 4D). CRISPR adaptation by both type I-E and I-F systems was reduced in the presence of RsmA (Figure 4D). In contrast to the type I systems, type III adaptation is not well understood and we are yet to detect adaptation under laboratory conditions. Therefore, we could not assess the effects of altered type III *cas1* and *cas2* levels on adaptation in *Serratia* (Figure 1D). In conclusion, the Rsm pathway, via RsmA, represses the type I adaptation genes, consequently decreasing spacer acquisition.

### RsmA represses type I CRISPR–Cas interference in other bacteria genera

Since the Rsm pathway is widespread in many bacteria and was controlling CRISPR–Cas in *Serratia*, we tested whether similar regulation occurs in a different genus. *Pectobacterium atrosepticum* SCRI1043 has a type I-F CRISPR–Cas system and genes encoding the Rsm pathway (62,63). We tested type I-F interference by assessing conjugation efficiency of a plasmid targeted by spacer 1 in the CRISPR1 array of *P. atrosepticum* compared with an untargeted control plasmid. As predicted, the untargeted plasmid conjugated efficiently into both the WT and a mutant disrupted for RsmA production (Supplementary Figure S5). The WT elicited interference against the targeted plasmid. Similar to the results with *Serratia*, we observed a significant ∼10-fold increase in interference in the *rsmA* mutant compared with the WT. Therefore, Rsm regulation of CRISPR–Cas immunity occurs in other genera.

### RsmA binds to CRISPR–Cas transcripts

**Figure 5.**
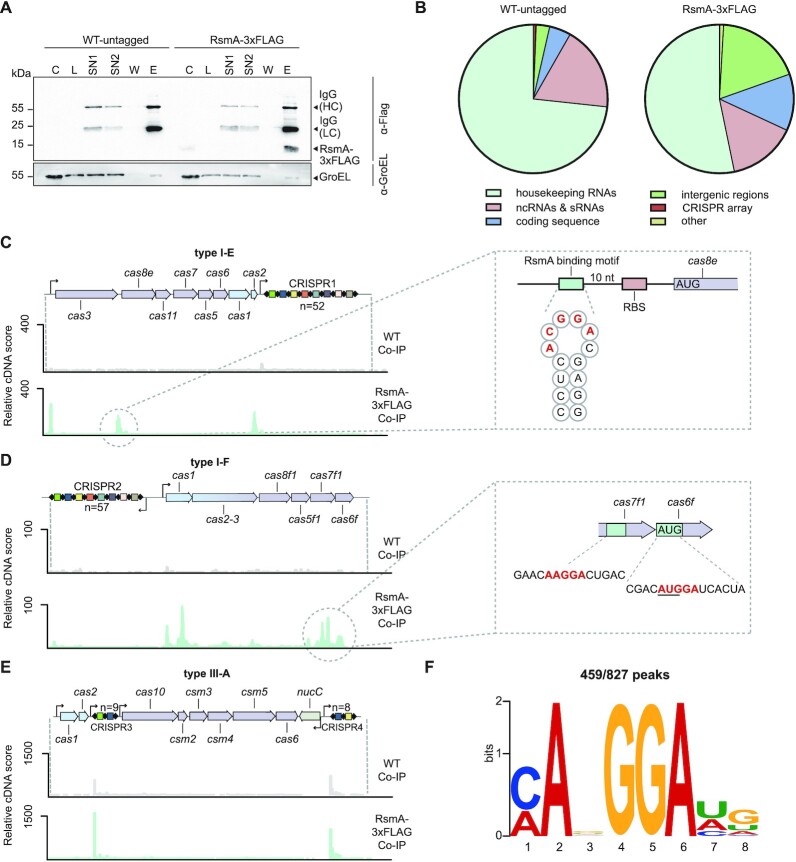
RsmA binds to type I *cas* mRNAs. (**A**) Western blot of Co-IP samples from WT and RsmA-3xFLAG strains using an anti-FLAG antibody confirms that RsmA-3xFLAG has been purified from the tagged strain. Samples were loaded per the OD_600_ of *Serratia* as indicated with GroEL used as control. C = culture protein, L = lysate, SN1 = supernatant 1, SN2 = supernatant 2, W = wash and E = elution fractions. (**B**) The relative proportions of RNA-seq reads of different RNA classes in the WT and RsmA-3xFLAG Co-IP libraries. RNA-seq reads mapping to the (**C**) type I-E, (**D**) type I-F and (**E**) type III-A CRISPR–Cas regions for the WT control (grey) and RsmA-3xFLAG Co-IP (green). Replicate 1 (R1) samples are shown for the top strand (no significant enrichment was observed on the bottom strand). Zoomed in regions show the predicted binding motif (red) with flanking nucleotides (black). (**F**) RsmA-binding motif predicted by MEME. See also Supplementary Figures S6, S7, S8 and Supplementary Tables S5, S6.

We have demonstrated that the Rsm pathway controls CRISPR–Cas but we did not know whether this regulation was direct or indirect. We hypothesised that RsmA directly affects CRISPR–Cas adaptive immunity by binding to *cas* mRNAs. To identify the specific genome-wide RNA targets directly bound by RsmA, we exploited RNA co-immunoprecipitation combined with deep sequencing (RIP-seq) (64). A chromosomal copy of *rsmA* was engineered with a C-terminal 3xFLAG epitope which allows for immunoprecipitation and isolation of native ribonucleoprotein complexes using an anti-FLAG antibody. Co-immunoprecipitation (Co-IP) was performed for both the RsmA-3xFLAG tagged and the WT untagged strains (Figure 5A) to compare and identify transcripts that are specifically bound to the RsmA-3xFLAG protein. The co-purified RNAs were converted to cDNA and deep sequenced. Approximately 94–95% of the ∼3.1–4.2 million reads per replicate library were mapped to the *Serratia* genome (Supplementary Table S5). Abundant cellular RNAs constitute ∼67% of reads from the untagged strain, which represents the background of non-specific binding (Figure 5B). In comparison, the RsmA-3xFLAG strain showed increased reads mapping to protein coding sequences (∼2.6-fold increase) and intergenic regions (∼6-fold increase) with a striking co-purification of RsmB (∼40-fold enrichment) (Figure 5B, Supplementary Table S6, Figure S6). We also identified enrichment of the *rsmA* transcript and other genes within the Rsm pathway, which suggests regulatory feedback that is proposed to enable rapid responses (Supplementary Figure S6) (23,24). As expected, transcripts previously shown to be regulated by RsmA in *Serratia*, such as the prodigiosin operon, were enriched by RsmA (29) (Supplementary Figure S7A, Supplementary Table S6).

We analysed whether RNAs originating from the CRISPR–Cas regions are specifically co-purified with the RsmA-3xFLAG protein (Figure 5C–E). There was a strong enrichment of type I-E and type I-F *cas* operons in RsmA-3xFLAG Co-IP samples (Figure 5C, D, Supplementary Table S6). In agreement with the expression data, enrichment peaks were observed in the 5′UTR of *cas3* (I-E), with further peaks identified within the *cas* coding sequences, in particular within *cas8e* and *cas2* from the type I-E and *cas1, cas2-3*, *cas7f1* and *cas6f* from the type I-F systems (Figure 5C, D and Supplementary Table S6). In agreement with our RNA-seq analysis, which showed that RNA levels from the non-coding CRISPR arrays were not significantly altered (Figures 1D, 3E, F), no enrichment in type I CRISPR RNAs by RsmA was detected (Figure 5C, D and Supplementary Table S6). We also did not detect enrichment in type III-A *cas* gene mRNAs by RsmA (Figure 5E). However, abundant non-specific RNA from the type III-A CRISPR3 and CRISPR4 loci were present in the control Co-IP in both replicates (Figure 5E, Supplementary Figure S7B and Supplementary Table S6). As the type III system in *Serratia* is contained within an integrative and conjugative element (ICE) (29), it is feasible that it is under indirect RsmA control, due to a more recent acquisition than the type I systems. In summary, Rsm-mediated control of type III-A CRISPR–Cas might occur indirectly via other intermediate regulators, whereas type I *cas* mRNAs are bound by RsmA.

### Enriched type I *cas* transcripts contain RsmA motifs

To characterise the binding motif for RsmA in *Serratia* we used an automated peak calling algorithm to identify and extract RNA regions specifically enriched by RsmA-3xFLAG. A conserved binding motif was identified in 459 of 827 peaks analysed using MEME (ANGGA; *e*-value = 2.8 × 10^−20^) (Figure 5F). This binding motif is consistent with an optimal *E. coli* CsrA site determined through *in vitro* binding and selection (AR**GGA**U; core binding motif in bold) (65). We searched for the ANGGA RsmA motif within the CRISPR–Cas regions where an enrichment of RNA binding by RsmA was observed.

Within the type I-E region there were three RsmA motifs in the 5′UTR of *cas3* (Figure 5C, Supplementary Figure S8). In agreement, this RsmA-enriched region and motifs were present in our *cas3*-zsGreen reporter, which was repressed by RsmA (Figure 3C). Another RsmA binding site is located 5′ of *cas8e* in a loop region of a predicted RNA hairpin and adjacent to a GGA within the RBS, suggesting a potential mechanism of translational control (Figure 5C, Supplementary Figure S8). We also detected a strong enrichment in type I-E *cas2* mRNA and three RsmA motifs were present within this gene. Since *cas2* is the final gene of the type I-E operon and involved in the adaptation step, these RsmA binding sites might explain the stronger effect of the Rsm pathway on type I-E adaptation when compared with interference (Figure 3). These data suggest direct control of the I-E *cas* operon by RsmA, but do not rule out the possibility of additional indirect effects.

Within the type I-F system, two RsmA motifs within the enriched RNA regions were present in the *cas1* coding sequence, one near the 5′ of the gene and another near the 3′ (Figure 5D and Supplementary Figure S8). Other RsmA motifs occurred near the middle of *cas7f1* and one site perfectly overlapped the ATG initiation codon of *cas6f*, consistent with a role in altering translation of these Cas proteins (Figure 5D). RNA-seq of the type I-F operon showed that the *rsmA* mutation caused an increasing amount of mRNA towards the 3′ end of the polycistronic transcript when compared with the WT (i.e. higher mRNA from genes towards the end of the operon) (Figure 3F). The considerable RsmA binding towards the 3′ end of the operon transcript (Figure 5D) might not only inhibit translation, but could lead to mRNA instability or aid Rho loading to increase transcriptional termination as observed for *E. coli glg* and *pga*, respectively (66,67). All of the predicted type I-F RsmA motifs were absent in the *cas1*-zsGreen reporter, which still displayed RsmA-dependency, suggesting additional indirect Rsm-mediated regulatory control of the type I-F operon. In combination, our data demonstrate that the RsmA-mediated regulation of the type I-E and I-F systems involves multiple binding sites throughout the *cas* transcripts.

### Phage sensitivity due to *rsmA* mutation is rescued by CRISPR immunity and some phages harbour *rsmA*

**Figure 6.**
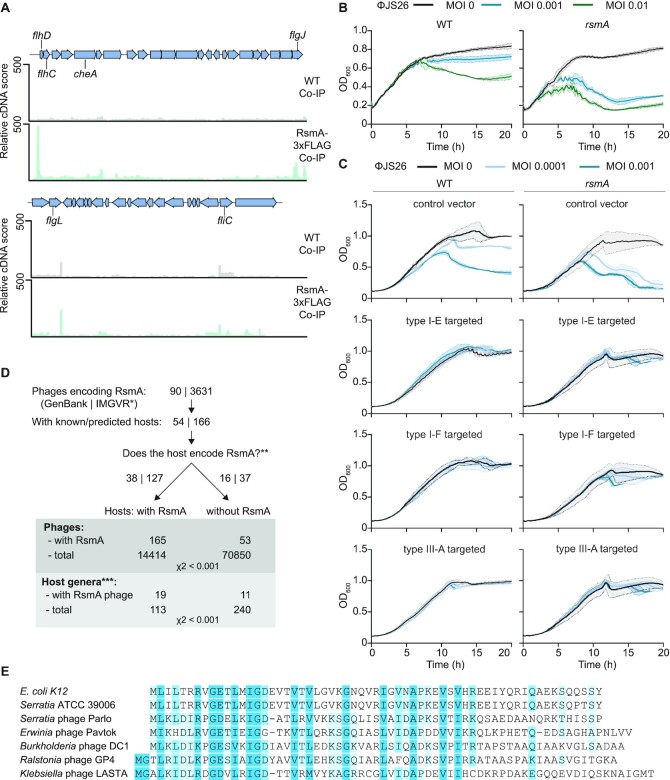
Mutation of *rsmA* results in sensitivity to phage infection, but is overcome by CRISPR–Cas immunity. (**A**) Mapping of the Co-IP RNA-seq reads demonstrates direct binding of RsmA to flagella loci, including the mRNA of the *flhDC* master regulator. The WT control (grey) and RsmA-3xFLAG Co-IP (green) are shown. Replicate 1 (R1) samples are shown for the top strand (no significant enrichment was observed on the bottom strand). (**B**) The *rsmA* mutant has increased sensitivity to phage JS26 compared with the WT strain (*n* = 3 biologically independent samples). (**C**) CRISPR immunity conferred by the type I-E, type I-F and type III-A CRISPR–Cas systems provides phage resistance to both the WT and *rsmA* mutant strains. In graphs, lines represent the mean ± the standard deviation. MOI = multiplicity of infection. (**D**) Bioinformatic identification of phages encoding RsmA. *For the IMG/VR dataset, only viral assemblies with an estimated completeness of >80% were searched. **Inference of the hosts encoding CsrA/RsmA is based on whether the corresponding host genus typically encodes CsrA/RsmA. ***Only host genera with five or more sequenced phages or viral contigs in the dataset are included. (**E**) Sequence comparison of select CsrA/RsmA homologs from bacteria and phages. See also Supplementary Figure S9.

One major function of the Rsm pathway is regulating the transition between sessile and motile states in response to metabolic cues (23). In *Serratia*, RsmA represses flagella-mediated swarming via the FlhDC master regulators of flagella biosynthesis (27–29). Accordingly, direct RsmA-mediated control of flagella synthesis was detected as a strong enrichment in the *flhDC* and other flagellar mRNAs by RIP-seq (Figure 6A, Supplementary Figure S8, Supplementary Table S6). Surprisingly, of the phages isolated that infect *Serratia* sp. ATCC 39006, almost all utilise flagella as a receptor (20,68). This led us to speculate that, by controlling flagella and CRISPR–Cas, the Rsm pathway promotes adaptive immunity when heightened motility would lead to elevated receptor availability and increased sensitivity to flagella-tropic phages. Conversely, free RsmA will repress flagella (27), rendering cells surface-immune to flagella-tropic phages and, as such, CRISPR–Cas immunity is concomitantly downregulated. To investigate if RsmA influences CRISPR-independent immunity, the flagella-tropic phage JS26 (69) was used to infect the WT and the *rsmA* mutant in the absence of CRISPR immunity. Consistent with increased flagella in *rsmA* mutants (27), the *rsmA* mutant was more sensitive to phage JS26 than the WT (Figure 6B). We reasoned that in the presence of CRISPR immunity, the increased sensitivity to phages in the *rsmA* mutant would be alleviated. To test this, we infected the WT and the *rsmA* mutant with phage JS26 in the presence of immunity provided by each of the three CRISPR–Cas systems (Figure 6C). In each case, the increased sensitivity of the *rsmA* mutant to JS26, presumably due to more phage receptor, was rescued by CRISPR immunity (Figure 6C). Therefore, when the Rsm pathway leads to increased sensitivity to a flagella-tropic phage, adaptive immunity can counteract phage sensitivity.

The selective pressure of CRISPR–Cas systems has led to the evolution of phage-encoded anti-defence strategies, such as anti-CRISPRs (70,71). We hypothesised that, since RsmA represses CRISPR–Cas systems, mobile genetic elements may exploit this by encoding similar proteins. We searched phage genomes in the GenBank and Integrated Microbial Genome/Virus (IMG/VR) databases for RsmA/CsrA homologs and discovered many examples of phage-encoded RsmA proteins (Figure 6D). For phages with known or predicted hosts, we then examined whether the corresponding bacteria encode their own RsmA/CsrA proteins, which revealed a significant correlation between the presence of phage and host RsmA/CsrA (χ^2^ < 0.001). To reduce potential biases due to similar phage sequences or overrepresentation of phages infecting specific genera, we also examined the data at the host genus level, finding a similar correlation between the presence of RsmA/CsrA in phages and hosts (χ^2^ < 0.001). Phage-encoded RsmA/CsrA were highly similar in sequence to bacterial RsmA/CsrA (Figure 6E). A recent bioinformatic analysis of RsmA/CsrA family proteins supports and extends our findings by demonstrating that homologues are not only present in phages, but are also carried by prophages, plasmids and integrative and conjugative elements (72). These RsmA/CsrA family proteins in mobile elements belong to different clusters and seem to have been acquired independently from bacteria multiple times (72). These bioinformatic analyses are supported by earlier studies showing RsmA-like proteins on a plasmid in *Sinorhizobium meliloti* and on integrative conjugative elements in *Legionella pneumophila* (73,74). Furthermore, the *S. meliloti* plasmid-encoded RsmA was functionally exchangeable with RsmA in *Pseudomonas fluorescens* (74). Taken together, we propose that mobile genetic elements have acquired host RsmA proteins, which may manipulate their bacterial hosts by regulating various cellular processes including CRISPR–Cas immunity.

## DISCUSSION

Here we demonstrated that the Rsm pathway controls the three CRISPR–Cas adaptive immune systems (I-E, I-F and III-A) in *Serratia*. The post-transcriptional regulator RsmA binds to type I-E and I-F transcripts in 5′UTRs and within coding sequences of these operons, and decreases *cas* gene expression, interference and adaptation. RsmA binding to these mRNAs likely influences translation and/or mRNA stability because increased *cas* gene mRNA levels occur in an *rsmA* mutant. The type III-A *cas* genes are also repressed by RsmA; however, this might occur indirectly through other pathways shown to affect type III expression (20). The CRISPR arrays of type I and III systems were unaffected by RsmA, consistent with the non-coding nature of these RNAs and their coupled processing and loading into CRISPR–Cas complexes. RsmA is the dominant component of the pathway and is sequestered by its antagonistic ncRNA, RsmB. In turn, RsmB is activated by the GacAS-family two-component system (PigW/PigQ) and antagonised by the membrane protein PigX (CsrD). We also demonstrated that RsmS acts independently of RsmB to control RsmA-dependent *cas* expression. Therefore, through the Rsm pathway, the activity of multiple CRISPR–Cas systems is being controlled post-transcriptionally by an RNA binding protein (RsmA) and a ncRNA (RsmB).

The Rsm pathway is a major regulator of bacterial behaviour in response to carbon sources (24). For example, in *E. coli*, the BarA/UvrY two-component system is activated by small carboxylic acids, such as formate and acetate, which are end products of glucose metabolism (75). These acids accumulate when carbon sources are scarce, a likely situation at high cell density (76) when bacterial populations are at risk of a phage epidemic (18,77). Furthermore, RNaseE-dependent CsrB/C turnover in *E. coli* is stimulated in response to glucose transport due to an interaction between unphosphorylated EIIA^Glc^ and CsrD (45). The net result of this pathway suggests that CRISPR–Cas activity will be enhanced when bacterial populations are reaching a high density upon exhausting preferred carbon sources. This may enable the integration of quorum sensing signalling with metabolic status to fine-tune adaptive immunity. The cAMP receptor protein (CRP), which is also involved in carbon utilisation, regulates CRISPR–Cas systems in *E. coli*, *Serratia, P. atrosepticum* and *Thermus thermophilus* (20,78–81), and is connected to the Csr pathways (82). Therefore, in *Serratia*, CRP might integrate further information into the Rsm pathway to control adaptive immunity.

The Rsm pathway plays an important role in controlling motility in *Serratia* (26–29), which can alter the vulnerability of cells to flagella-tropic phages, such as JS26. We propose that by simultaneously upregulating motility and CRISPR–Cas, the Rsm pathway may enable bacteria to offset the risks associated with elevated phage surface receptors through enhanced adaptive immunity. Conversely, when free RsmA is elevated, flagella and CRISPR–Cas are repressed, with cell surface immunity rendering CRISPR–Cas superfluous. Given the role of the Csr pathway in altering cellular behaviour in response to metabolic status, it is likely that the Rsm pathway uses similar signals to control CRISPR–Cas and motility in *Serratia*. We recently discovered similar coordinate control of surface-based phage immunity and CRISPR–Cas by the Rcs envelope stress response phosphorelay (20). Interestingly, a recent study in *Salmonella* discovered that SirA (PigQ in *Serratia*) activated the *rcsDB* operon and that BarA (*Serratia* PigW) could phosphorylate the RcsB transcriptional regulator (83). We have also previously shown that *pigQ* transcription is quorum sensing-dependent (25). Therefore, it appears that multiple regulatory pathways are interconnected and converge to control CRISPR–Cas in response to different signals and stresses (10,11).

The importance of RsmA as an inhibitor of three different CRISPR–Cas systems was supported by the discovery of RsmA/CsrA proteins in multiple phages, and suggested that these virally-encoded proteins might have been selected to suppress bacterial defences. RsmA/CsrA homologues have been previously identified in phages; for example, *Burkholderia* phage DC1 (84) and the *P. aeruginosa* F116 transducing phage (85). Furthermore, plasmids and genomic islands have independently acquired RsmA-family proteins (72–74). In one case, a mobile Rsm protein could regulate cellular Rsm-dependent pathways in a different genus (74), supporting our proposal that mobile elements have acquired RsmA-family proteins to control bacterial physiology, including CRISPR–Cas immunity. Due to the pleiotropic nature of the Rsm pathway, mobile RsmA-family proteins are also likely to be involved in broad-scale manipulation of the bacterial host during infection to favour invader replication (72). In fact, *csrA* was engineered into an M13 filamentous phage, which led to manipulation of biofilm and antibiotic sensitivity properties of *E. coli* (86). Our work adds to the concept that regulatory proteins in phage genomes may have anti-CRISPR activity (71). Indeed, a regulator of alginate biosynthesis in *P. aeruginosa* was recently shown to be encoded by some phages and repress CRISPR–Cas expression (87).

The existence of RsmA homologues encoded by multiple phages and other mobile elements indicates that RsmA is likely to control CRISPR–Cas in other bacteria. Indeed, we demonstrated that RsmA affects interference by the *P. atrosepticum* type I-F CRISPR–Cas system. Furthermore, in an RNA-seq study, type I-E *cas* genes were elevated in a *csrA* mutant of *Erwinia amylovora* (88). During the preparation of our manuscript, a calcium-responsive kinase was shown to act via RsmA to control type I-F CRISPR–Cas in *P. aeruginosa* (89). Taken together, regulation of CRISPR–Cas adaptive immunity by the Rsm pathway is not restricted to *Serratia* and occurs more widely in different bacterial genera. The presence of RsmA/CsrA homologues in some Gram-positive bacteria, raises the further possibility that CRISPR–Cas control by these proteins might be even broader (90).

Unlike most CRISPR–Cas regulation characterised thus far, the Rsm pathway is based on an RNA binding protein that provides post-transcriptional control. Post-transcriptional control can enable faster responses, since release of translational inhibition can result in protein production independently of nascent transcription. Because CRISPR–Cas regulation acts both transcriptionally and post-transcriptionally, we predict this enables both tighter and integrated control of adaptive immunity.

## DATA AVAILABILITY

The RNA-seq and RIP-seq data in this publication have been deposited in NCBI’s Gene Expression Omnibus (91) and are accessible through GEO Series accession number GSE161713.

## Supplementary Material

gkab704_Supplemental_Files
