# Supplementary material for: The Rsm (Csr) post-transcriptional regulatory pathway coordinately controls multiple CRISPR–Cas immune systems
Source: Nucleic Acids Res. 2021 Aug 17;49(16):9508–25. doi: 10.1093/nar/gkab704 (PMC8450108; doi:10.1093/nar/gkab704)
Supplement: gkab704_Supplemental_Files [file gkab704_supplemental_files.zip › Rey_Campa_etal_NAR_2021_Supplementary_R1.pdf]

SUPPLEMENTARY MATERIAL

The Rsm (Csr) post-transcriptional regulatory pathway coordinately controls multiple CRISPR-Cas immune systems

Aroa Rey Campa[1,2], Leah M. Smith[1,†], Hannah G. Hampton[1,†], Sahil Sharma[3], Simon A. Jackson[1,4], Thorsten Bischler[5], Cynthia M. Sharma[3] and Peter C. Fineran[1,2,4,*]

SUPPLEMENTARY TABLES

Table S1. List of strains used in this study.

Strain name	Description	References
Serratia sp. ATCC 39006 strains		
WT (LacA)	*Serratia* sp. ATCC 39006 Lac- derivative	(1)
NMW7	*rsmA*::Tn-DS1028*lacZ*Km, Km^{R}	(2)
NW64	*rsmA*::Tn-DS1028*uidA*Cm, Cm^{R}	(3)
PCF396	Pigmentless (Δ*pigA-O*) derivative of *Serratia* LacA	(4)
PCF398	*rsmA*::Tn-DS1028*lacZ*Km, Δ*pigA-O*, Km^{R}	(4)
PCF406	*rsmB*::mini-Tn*5lacZ*1, Δ*pigA-O*, Km^{R}	(4)
PCF629	*pigQ*::mini-Tn*5*Sm/Sp, Δ*pigA-O*, Sp^{R}	This study
PCF630	*pigW*::mini-Tn*5*Sm/Sp, Δ*pigA-O*, Sp^{R}	This study
PCF631	*pigX*::mini-Tn*5*Sm/Sp, Δ*pigA-O*, Sp^{R}	This study
PCF694	*rsmS::*Tn-DS1028*uidA*Cm, Δ*pigA-O*, Cm^{R}	This study
PCF703	*rsmA*::Tn-DS1028*lacZ*Km, *rsmS*::Tn-DS1028*uidA*Cm, Δ*pigA-O*, Km^{R}, Cm^{R}	This study
PCF675	*rsmA*::Tn-DS1028*uidA*Cm, *rsmB*::mini-Tn5*lacZ*1, Km^{R}, Cm^{R}	This study
PCF704	*rsmA*::Tn-DS1028*uidA*Cm, *pigX*::mini-Tn*5*Sm/Sp, Δ*pigA-O*, Cm^{R}, Sp^{R}	This study
PCF705	*rsmS*::Tn-DS1028*uidA*Cm, *rsmB*::mini-Tn5*lacZ*1, Δ*pigA-O*, Cm^{R}, Km^{R}	This study
PCF706	*rsmS*::Tn-DS1028*uidA*Cm, *pigX*::mini-Tn*5*Sm/Sp, Δ*pigA-O*, Cm^{R}, Sp^{R}	This study
PCF717	*rsmB*::*uidA*Cm, *pigX*::mini-Tn5Sm/Sp, Δ*pigA-O*, Cm^{R}, Sp^{R}	This study
PCF624	*rsmA*-3xFLAG (C-terminal)	This study
Pectobacterium atrosepticum SCRI1043 strains		
WT	*Pectobacterium atrosepticum* SCRI1043	(5)
AE9	*rsmA*::mini-Tn*5*Sp/Sm	(6)
Escherichia coli strains		
ST18	Conjugative donor: *pro,hsdR, recA*::RP4-2-Tc::Mu, Tmp^{R}, Sp^{R}, Sm^{R}, λ*pir* Δ*hemA*	(7)
DH5α	Cloning strain: F^{-}, ϕ80Δd*lacZ*M15, Δ(*lacZYA–argF*)U169, *endA1, recA1, hsd*R17 (r_K^- m_K^+), *deoR*, *thi*-1, *sup*E44, λ^{-} , *gyr*A96, *rel*A1	Gibco/BRL

Table S2. List of plasmids used in this study.

Plasmid	Description	Construction notes	Reference
Interference			
pPF719	Untargeted control for type I, pQE80L-oriT, pBR322/ori, RP4/oriT, lacI/T5, Tc^R		(8)
pPF724	Targeted type I-E CRISPR1 spacer 1 (CTT PAM) pQE80L-oriT, pBR322/ori, RP4/oriT, lacI/T5, Tc^R		(8)
pPF722	Targeted type I-F CRISPR2 spacer 1 (GG PAM) pQE80L-oriT, pBR322/ori, RP4/oriT, lacI/T5, Tc^R		(8)
pPF781	Untargeted control for type III-A, pBAD, p15A/ori, RP4/oriT, araC, Cm^R		(8)
pPF1043	Targeted type III-A with spacer 1 CRISPR3, pBAD, p15A/ori, RP4/oriT, araC, Cm		(8)
pPF571	Untargeted control plasmid, pQE-80L-oriT-mCherry-derivative, pBR322/ori, RP4/oriT, lacI/T5, Tc^R		(9)
pPF572	*P. atrosepticum* CRISPR1 spacer 1 targeting plasmid, pPF571 derivative, pBR322/ori, RP4/oriT, lacI/T5, Tc^R		(10)
Adaptation			
pPF953	Naïve priming plasmid, RP4/oriT, lacI/T5, mCherry, Tc^R		(11)
pPF1233	Priming plasmid I-E, with protospacer CRISPR1 spacer 1 (CGT_PAM), RP4/oriT, lacI/T5, mCherry, Tc^R		(4)
pPF1236	Priming plasmid I-F, with protospacer from CRISPR2 spacer 2 (AGA_PAM), RP4/oriT, lacI/T5, mCherry, Tc^R		(4)
rsmA-3xFLAG			
pPF1117	Suicide vector for allelic exchange mutagenesis. Cm^R, R6K, RP4/oriT, sacB		(11)
pPF1811	C-terminal 3xFLAG-tag *rsmA* suicide vector, pPF1117 derivative, Cm^R, R6K, oriT, sacB	pPF1117 + PF3562 digested with BamHI and SacI	This study
Reporters			
pPF1300	Entry vector with pBR322/ori, RP4/oriT, MCS, Cm^R		(4)
pPF1382	Entry vector with pBR322/ori, RP4/oriT, MCS, Gm^R	Ligate Gm^R fragment (PF2257 (EcoRI) PF2558 (NcoI)) from p34S-Gm; with PCR pPF1300 outward from Cm^R (PF2259 (NcoI) PF2560 (EcoRI).	This study
pPF1847	Entry vector + MCS with pBR322/ori, RP4/oriT, MCS, Gm^R	pPF1300 + annealed PF2225 + PF2226 digested with XmaI and MfeI	This study
pPF1849	Entry vector + MCS + terminator with pBR322/ori, RP4/oriT, MCS, Gm^R	pPF1847 + PCR product PF3122 + PF3570 digested with HindIII and SphI	This study
pPF1854	zsGreen reporter vector, pBR322/ori, RP4/oriT, MCS, Gm^R	pPF1849 + PCR product PF3579 + PF3580 in pPF1706 template,	This study

Plasmid	Description	Construction notes	Reference
		digested with BamHI and NheI	
pPF1890	*cas1*-zsGreen reporter (type I-F), pBR322/ori, RP4/oriT, MCS, Gm^R	pPF1854 + PF3644-PF3643 digested with SpeI and NsiI	This study
pPF1891	*cas10*-zsGreen reporter (type III-A), pBR322/ori, RP4/oriT, MCS, Gm^R	pPF1854 + PF3641-PF3642 digested with SpeI and NsiI	This study
pPF1973	*cas3*-zsGreen reporter (type I-E), pBR322/ori, RP4/oriT, MCS, Gm^R	pPF1854 + PF3696-PF3852 digested with SpeI and NsiI	This study
pPF1976	*rsmB*-zsGreen reporter vector promoter, pBR322/ori, RP4/oriT, MCS, Gm^R	pPF1854 + PF4061-PF4062 digested with SpeI and NsiI	This study
Complementation			
pPF781	pBAD vector with p15A/ori, RP4/oriT, araC, Cm^R		(8)
pPF1958	RsmA expression vector, p15A/ori, RP4/oriT, araC, Cm^R	pPF781 + PF788-PF789 digested with EcoRI and HindIII	This study
pPF1959	RsmB expression vector p15A/ori, RP4/oriT, araC, Cm^R	pPF781 + PF3568-PF3569 digested with EcoRI and HindIII	This study
pPF1960	PigQ expression vector, p15A/ori, RP4/oriT, araC, Cm^R	pPF781 + PF3143-PF3144 digested with EcoRI and HindIII	This study
pPF1961	PigW expression vector, p15A/ori, RP4/oriT, araC, Cm^R	pPF781 + PF3147-PF3148 digested with EcoRI and HindIII	This study
pPF1962	PigX expression vector, p15A/ori, RP4/oriT, araC, Cm^R	pPF781 + PF3145-PF3146 digested with EcoRI and HindIII	This study
pPF1964	RsmS expression vector, p15A/ori, araC, Ap^R	pBAD30 + PF810-PF811 digested with EcoRI and HindIII	This study
pQE-80LoriT	pQE-80L derivative with pBR322/ori, RP4/oriT, Ap^R		(12)
pPF513	RsmA expression vector, pBR322/ori, RP4/oriT, Ap^R	pQE-80LoriT + PF788-PF789 digested with EcoRI and HindIII	This study
Phage assays			
pPF260	pQE-80L derivative with pBR322/ori, RP4/oriT, Km^R		(9)
pPF1473	Anti-JS26 type III-A spacer, pBR322/ori, RP4/oriT, Km^R		(13)
pPF1485	Anti-JS26 type I-E spacer, pBR322/ori, RP4/oriT, Km^R		(13)
pPF1489	Anti-JS26 type I-F spacer, pBR322/ori, RP4/oriT, Km^R		(13)

Table S3. Oligonucleotides used in this study.

Primer name	Description	Sequence
Expression plasmids		
PF788	Fw RsmA	ATA**GAATTC**AGGAGGAATATAATGCTTATTTTAACTCGTCG
PF789	Rv RsmA	GAT**AAGCTT**TCAATAAGATGTTGGCTGAG
PF3568	Fw RsmB	TTT**GAATTC**GTGTAAGTGGCTTGCCGATTCAG
PF3569	Rv RsmB	TTT**AAGCTT**GATCACTCAACAGCCAATTCTGACGG
PF3143	Fw PigQ	TTT**GAATTC**AAGAGGAGAAATTAACTATGATTAGCGTTTTTC<br>TTGTTG
PF3144	Rv PigQ	TTT**AAGCTT**TCACTCACTGCTAATCAATGTC
PF3147	Fw PigW	TTT**GAATTC**AAGAGGAGAAATTAACTATGACCAAATACAGT<br>CTGCGTG
PF3148	Rv PigW	TTT**AAGCTT**TCAGTTGATTTTTGTCAGATGCC
PF3145	Fw PigX	TTT**GAATTC**AAGAGGAAATTAACTATGGGATTTACTGCAAA<br>AATTT
PF3146	Rv PigX	TTT**AAGCTT**TTAAACATAAGCACGAGATGAATAT
PF810	Fw RsmS	ATA**GAATTC**AGGAGGAATATAATGTCACTGGAAAATGCAGC
PF811	Rv RsmS	GAT**AAGCTT**TTATCTTGCCATGCTGTCTTC
Reporters		
PF2225	Fw MCS for pPF1170	GGCCCGGGTACCAATTGACTAGTCACGTCGTCGACATGCA<br>TG
PF2226	Rv MCS for pPF1170	GATCCATGCATGTCGACGACGTGACTAGTCAATTGGTACC<br>CGGGCCTGCA
PF3122	Fw terminator	TTT**CCCGGG**GACTCCTGTTGATAGATC
PF3570	Rv terminator	TTT**GCATGC**GCTTGGATTCTCACCAATA
PF3579	Fw zsGreen	TTT**GGATCC**GCACAGAGCAAACATGGACTG
PF3580	Rv zsGreen	TTT**GCTAGC**CATTACGGCAGTGCAGAACC
PF3696	Fw *cas3* (I-E)	TTT**ACTAGT**GTCTGGTCGAAGGTCGGTTG
PF3852	Rv *cas3* (I-E)	TTT**ATGCAT**AGTTAATTTCTCCTCTTCAAATGCGTTGGCTTT<br>CC
PF3644	Fw *cas1* (I-F)	TTT**ACTAGT**TTATTTGCACAAGCCACTTTTC
PF3643	Rv *cas1* (I-F)	TTT**ATGCAT**AATATATTCTCTGCTATCGCGATTAG
PF3641	Fw *cas10* (III-A)	TTT**ACTAGT**AGTAGGTACTATTTCTTTGG
PF3642	Rv *cas10* (III-A)	TTT**ATGCAT**TGACATCTCCTTGTGCC
PF4061	Fw *rsmB* promoter	TTT**ACTAGT**CTGCCTTGTAAGATATCTCTT
PF4062	Rv *rsmB* promoter	TTT**ATGCAT**AGTTAATTTCCTCTTATAAGATGTATTAATTAAA<br>CTGAATCG
Allelic exchange		
PF3562	gBlock RsmA-3xFLAG C-term	TCTTCACCTCGAGAAATC**GGATCC**TGGGGTAACCAAAACG<br>TTGACTGACAGAGTTAAGGCTGGCGAACTCATCGGATTCG<br>TGGCGAACCAGATTGGTGGCAAAGGTGGTGGTCGTCCTGA<br>TATGGCTCAGGCGGGCGGCAGTGATGTCAAGGCATTACCA<br>TCTGCGCTAGCCAGCGTTGAAGGCTGGGTGGCTAATAAGT<br>TATAAGTATTATTATACCAAGCGCTATCCTGGCAAAACGCC<br>ATGACTCTTAATGGTTTTGGCGTTTTTGGCCTGCGGTAAAG<br>GATGACCGTAAACAAGACGAATCGTGTGTCTCTTTCAGAAT<br>GTTGAATGCAGAATATGCCTTGGTCTGGTTGTGATAACAAA<br>AAACGCAAGCTGTCTTATATCGGCTAAACTTAACATTAGTA<br>ACAAGCTATGAGTGGGATGATGAGGCAATTATTGTCATCTA<br>GGTTTACGTTTCCTCGGCACATGATGGATAATGGCGAGAG<br>AACGAAGAGACCTGACTCTTTATAATCTTTCAAGGAGCAAA

Primer name	Description	Sequence
		GAATGCTTATTTTAACTCGTCGAGTTGGCGAAACCCTCATG ATTGGCGATGAGGTAACGGTTACCGTACTAGGAGTAAAGG GCAATCAGGTTCGTATTGGTGTGAATGCGCCTAAAGAAGTT TCTGTGCACCGCGAAGAGATTTACCAACGTATTCAGGCAG AGAAGTCTCAGCCAACATCTTATGACTACAAGGACCACGAC GGTGACTACAAGGACCACGACATCGACTACAAGGACGACG ACGACAAGTGATTCACAATGCGTCTCGCTCTCACGAGGCG CTATTGTTGTTCTGGTTTTTGATTCGAATTTTCAGCTCCCCT CATTTTTATTTTTTGCATTCACATCTTGTCTGATACAGGATA ATCAGTTGTTTTCCTGTTGATAATATATTCATTTTGTTGCCA ATCCATCCGTGTTGGGTGCGAATTGTGCAAACAAACATGA GAAGGGAAAAATTGTTTGACTTATAAGTCCTGGAAAGTAAT ATGTGCGCCACGCAGTATCGGTGAGCACTGAACGAGAAAT CAAAAAGTAATCATTGGTAGCGTAAGGTGAGGTGGCCGAG AGGCTGAAGGCGCTCCCCTGCTAAGGGAGTATGCGGTCAA AAGCTGCATCGAGGGTTCGAATCCCTCCCTCACCGCCATT AATATGCATCCGTAGCTCAGCTGGATAGAGTACTCGGCTA CGAACCGAGCGGTCGGAGGTTCGAATCCTCCCGGATGCA CCATTTAGTGGGTGTGTTTTGTCGG**GAGCTC**CCTGTTGATA GATCCAGT
Screening		
PF3563	Fw RsmA-3xFLAG	CGTATTCAGGCAGAGAAGTCTCAGC
PF3564	Rv RsmA-3xFLAG	CGCTCTCACGAGGCGCTATTG
PF3565	Rv RsmA-3xFLAG	CAATATGGTGCATCCGGGAGGATTC
PF2240	Fw pPF1300	CTCTGGCTCACCGACGAC
PF2242	Rv pPF1300	GCTCAGGAGAGCGTTCAC
PF796	Fw *flhDC* primer	ATAGAATTCAGGAGGAATATAATGGGTAC TTCTGAGTTACTTAAGC
PF797	Rv *flhDC* primer	GATCCCGGGTCAGACTGCGTGTTTTACTTG
Adaptation		
PF1887	Fw CRISPR1 (I-E)	GTTAAGTCAGCAGGCGTTTAGTCG
PF1989	Rv CRISPR1 (I-E)	TAAGTTAGTGTTCTTTAACAAGCAGGA
PF1888	Fw CRISPR2 (I-F)	CATCTGATGCTGACGACACTG
PF1990	Rv CRISPR2 (I-F)	CACGAAAATGATAATTGATGCTGAT

Restriction sites in **bold** and Ribosome binding sites underlined.

Table S4. RNA-seq results from DESeq2 of *Serratia* genes with in the *rsmA* mutant compared with the WT. See separate Supplementary Table S4 file (.xls).

Table S5. RIP-seq read mapping statistics against *Serratia*

Sample	Total reads	Aligned reads (%)	Multimapped reads (%)*	Unique reads (%)	Unmapped reads (%)
CoIP_WT_RsmA_R1	3144737	94%	60%	34%	6%
CoIP_WT_RsmA_R2	4214068	94%	66%	28%	6%
CoIP_WT_RsmA_3xFLAG_R1	4221559	95%	38%	57%	5%
CoIP_WT_RsmA_3xFLAG_R2	3777198	95%	58%	37%	5%

*Reads that map to multiple locations equally well (e.g. rRNA reads) were divided equally amongst all potential mapping locations

Table S6. List of *Serratia* genomic regions significantly enriched by RsmA-3xFLAG. See separate Supplementary Table S6 file (.xls).

SUPPLEMENTARY FIGURES

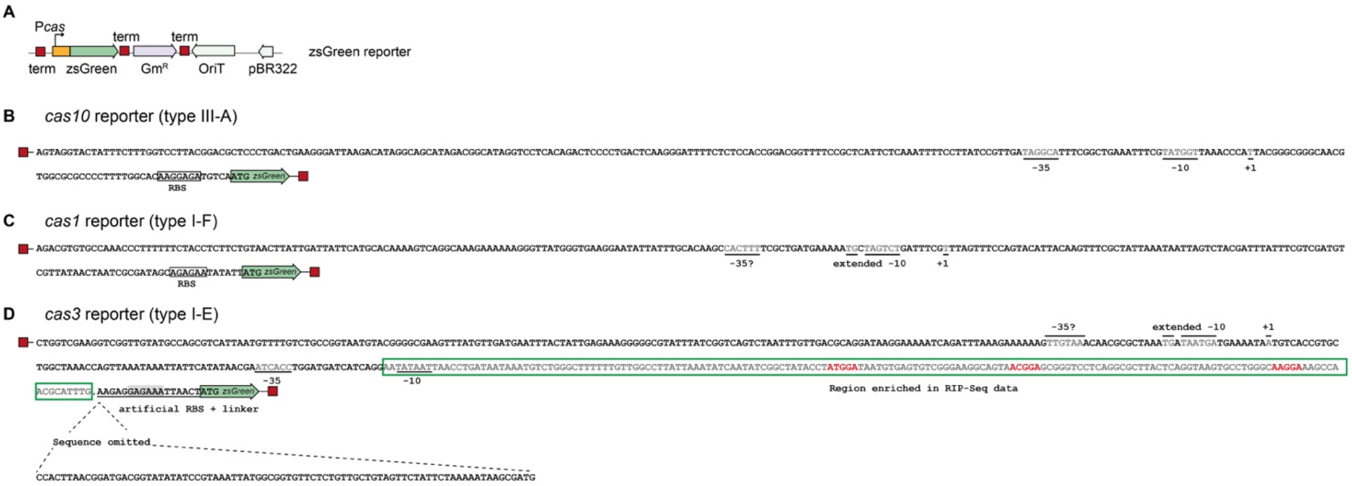

Figure S1. **Schematic of *cas* zsGreen reporters. A)** Schematic of zsGreen reporter plasmid used in this study. **B)** Type III-A *cas10* reporter plasmid (pPF1891). **C)** Type I-F *cas1* reporter plasmid (pPF1890). **D)** Type I-E *cas3* reporter plasmid (pPF1973). Boxed regions indicates natural ribosome binding sites (RBS), grey shading indicates vector RBS and green box shows the *cas3* 5'UTR region identified by RIP-seq as enriched by RsmA.

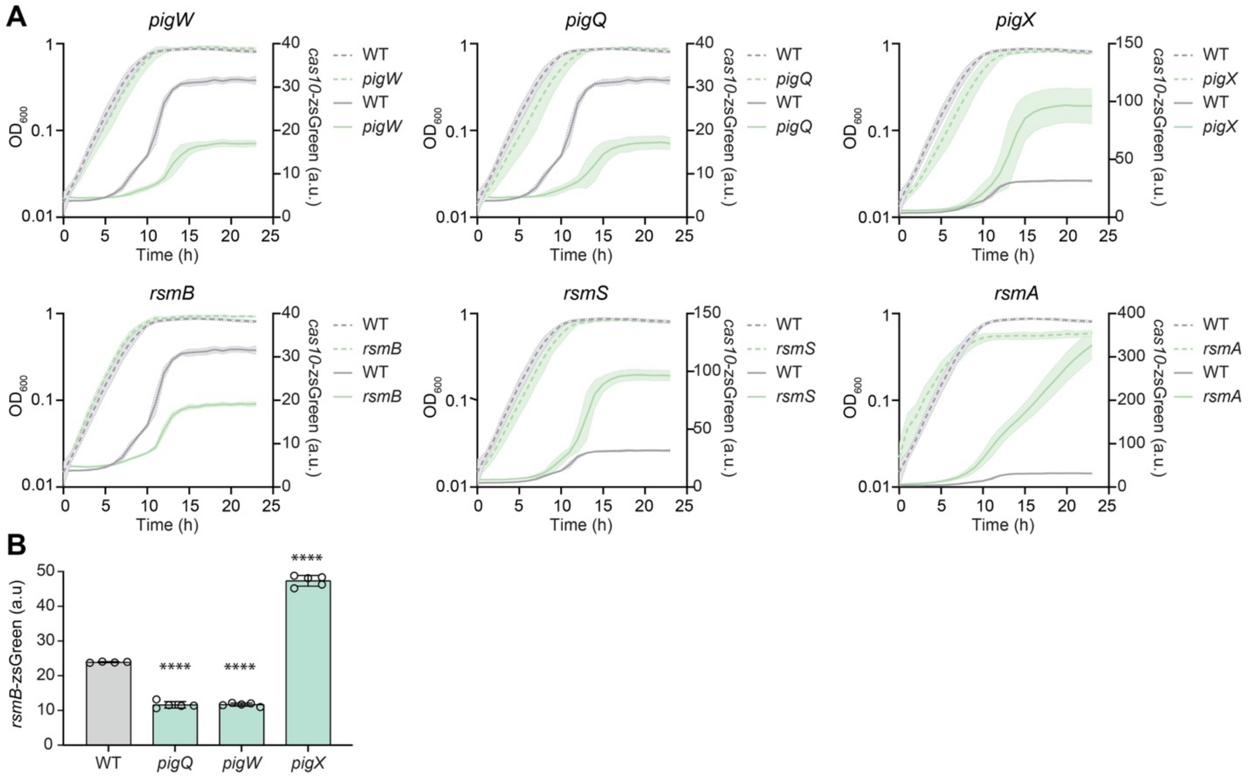

Figure S2. **The Rsm pathway controls type III-A CRISPR-Cas expression. A)** Expression of a *cas10* (*csm*) zsGreen reporter (III-A system) in different Rsm pathway mutants compared with the WT control (n=4 biologically independent samples). Lines represent the mean of the biological replicates and the shading represents ± standard deviation. Dotted lines represent OD_{600} and solid lines represent fluorescent measurements. **B)** The PigQW two component system and PigX control RsmB levels. Expression of an *rsmB* reporter in the WT, *pigW*, *pigW* and *pigX* mutant backgrounds (n≥4 biologically independent samples). All bars represent the mean and error bars represent the standard deviation. To determine statistical significance, two-sided *t*-tests were used (****P<0.0001). a.u., arbitrary units.

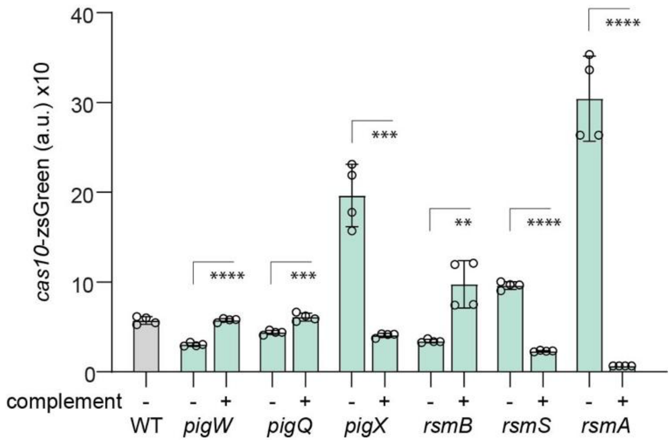

Figure S3. All members of the Rsm pathway complement their respective mutants for type III-A expression. Expression of a *cas10* (*csm*) zsGreen reporter in different Rsm pathway mutants compared with the WT control. Each Rsm pathway mutant contained a second complementing plasmid (+) or the empty vector control (-), and the WT control contained the empty vector. a.u., arbitrary units. (n=4 independent biological replicates). All bars represent the mean and error bars represent the standard deviation. To determine statistical significance, two-sided *t*-tests were used (****P<0.0001; ***P<0.001; **P<0.01).

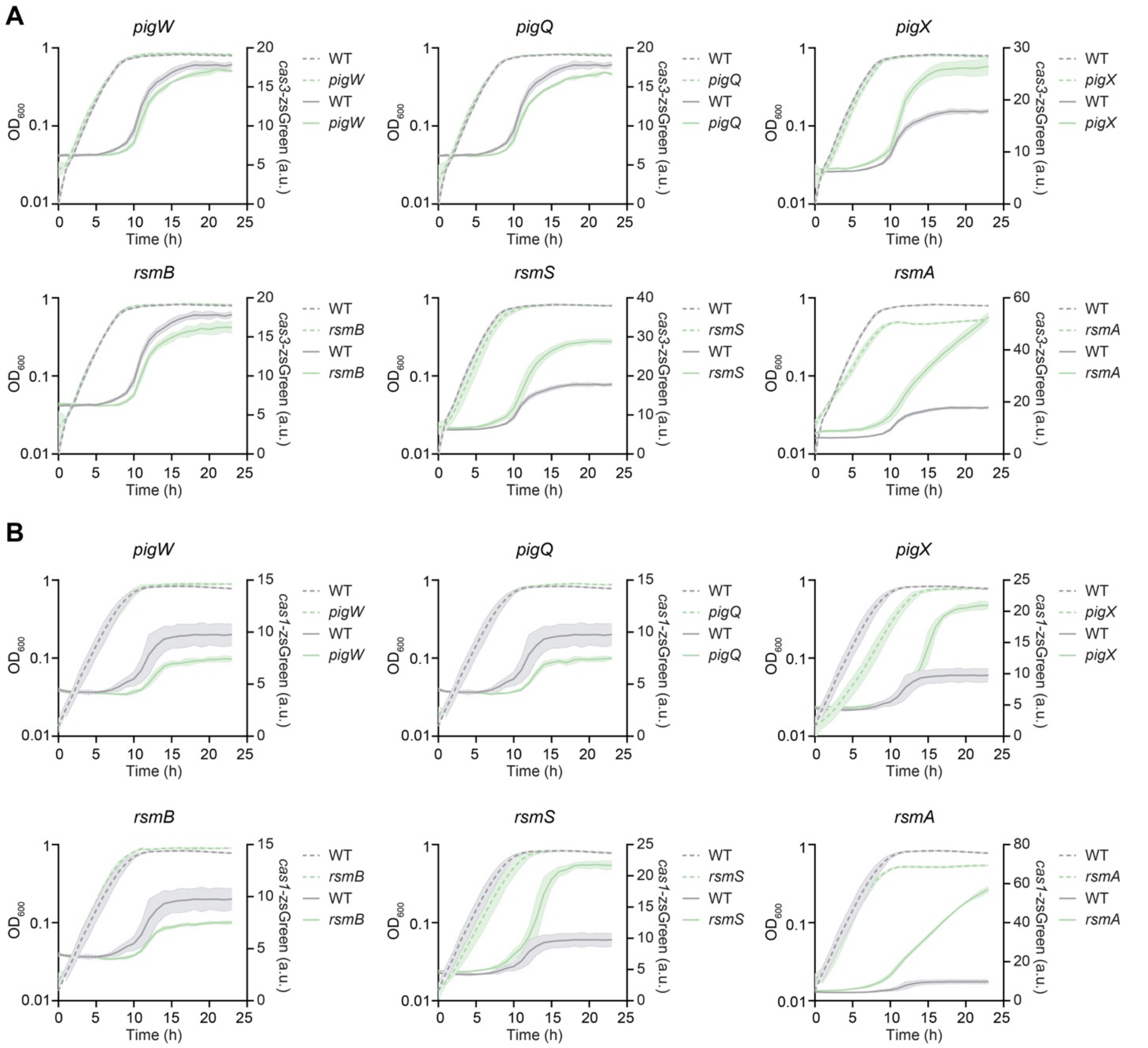

Figure S4. The Rsm pathway controls type I-E and I-F CRISPR-Cas expression. A) Expression of a *cas3*-zsGreen reporter (I-E system) in different Rsm pathway mutants compared with the WT control (n=4 biologically independent samples). **B)** Expression of a *cas1*-zsGreen reporter (I-F system) in different Rsm pathway mutants compared with the WT control (n=4 biologically independent samples). Dotted lines represent OD_{600} and solid lines represent fluorescent measurements. a.u., arbitrary units. Lines represent the mean of the biological replicates and the shading represents ± standard deviation.

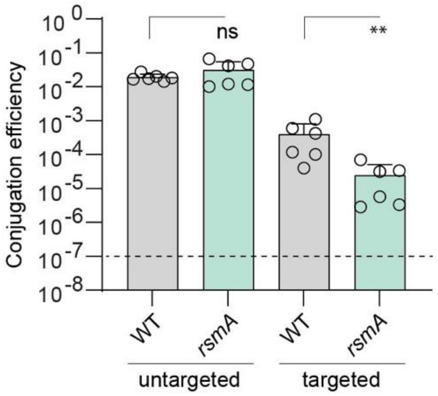

Figure S5. Interference by the *P. atrosepticum* type I-F CRISPR-Cas system is repressed by RsmA. Type I-F CRISPR-Cas interference in the WT and an *rsmA* mutant measured as conjugation efficiency of plasmids with a protospacer targeted by the CRISPR1 array (targeted) or a control lacking a protospacer (untargeted) (n=6 biologically independent samples). The dashed line indicates the limit of detection. All bars represent the mean and error bars represent the standard deviation. To determine statistical significance, two-sided *t*-tests were used on log-transformed data (**P<0.01; ns=not significant).

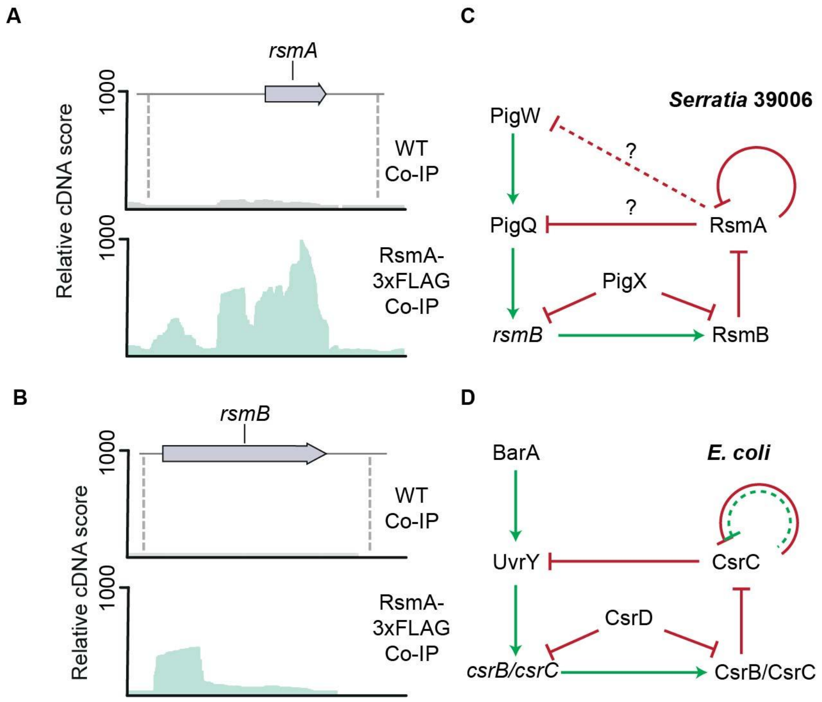

Figure S6. **Potential feedback regulatory loops in the Rsm pathway revealed by RIP-seq. A)** Clear enrichment around *rsmA* suggests that RsmA is autoregulated similarly to what is observed in *E. coli* (14). **B)** RsmB is enriched by RsmA. In (A) and (B) relative cDNA scores for the WT control are grey and RsmA-3xFLAG Co-IP is green. Replicate 1 (R1) samples are shown. **C)** Schematic of potential autoregulation of the Rsm pathway in *Serratia*. RsmA bound the *pigQ* (*uvrY*) mRNA and, to a lesser extent, *pigW* (*barA*) and *pigX* (*csrD*) transcripts, although these were not statistically significant (**Supplementary Table S5**). **D)** Schematic of the autoregulation of the Csr pathway in *E. coli*. Internal regulatory networks within the Rsm/Csr pathway also occur in other species, but can vary (14). These feedback loops and autoregulatory circuits are proposed to enable the Csr system to have rapid responses to signals (15).

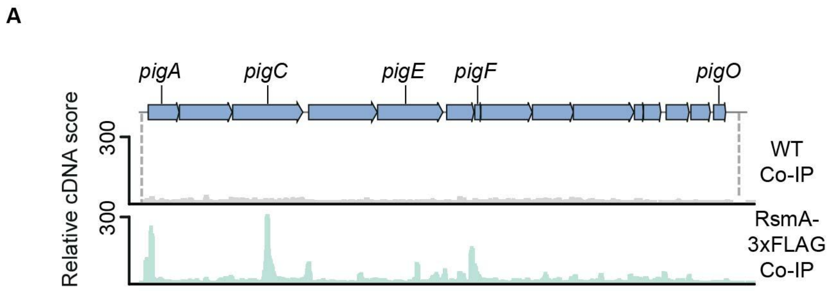

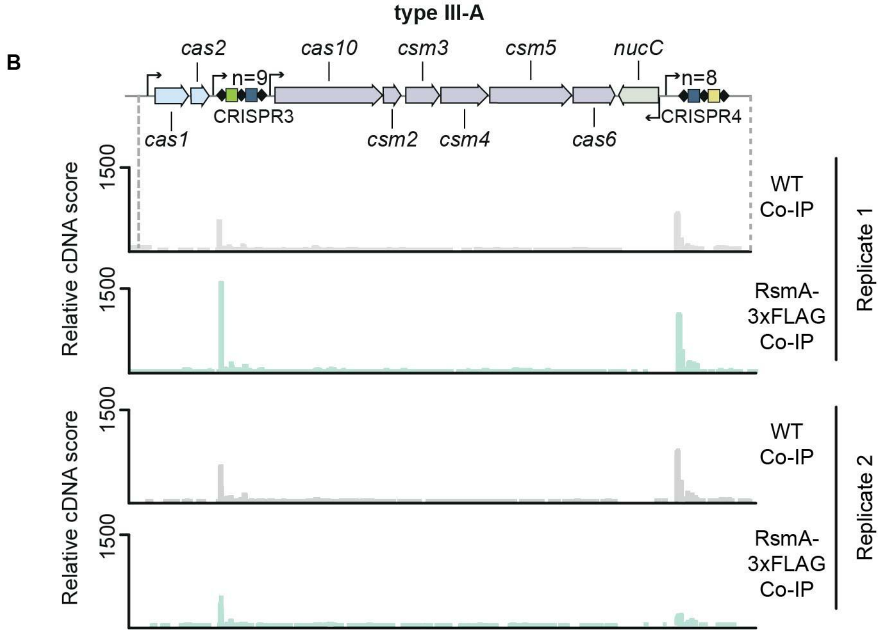

Figure S7. RsmA-3xFLAG binds to mRNAs known to be under RsmA control and type III CRISPRs are non-specifically purified. RIP-seq reads mapping to the **A)** prodigiosin (*pigA-O*) operon region for the WT control (grey) and RsmA-3xFLAG Co-IP (green). Replicate (R1) is shown. **B)** Figure 5 showing both replicates (R1 and R2), which highlights the non-specific purification of CRISPR arrays. Complete RIP-seq data is provided in **Supplementary Table S5**.

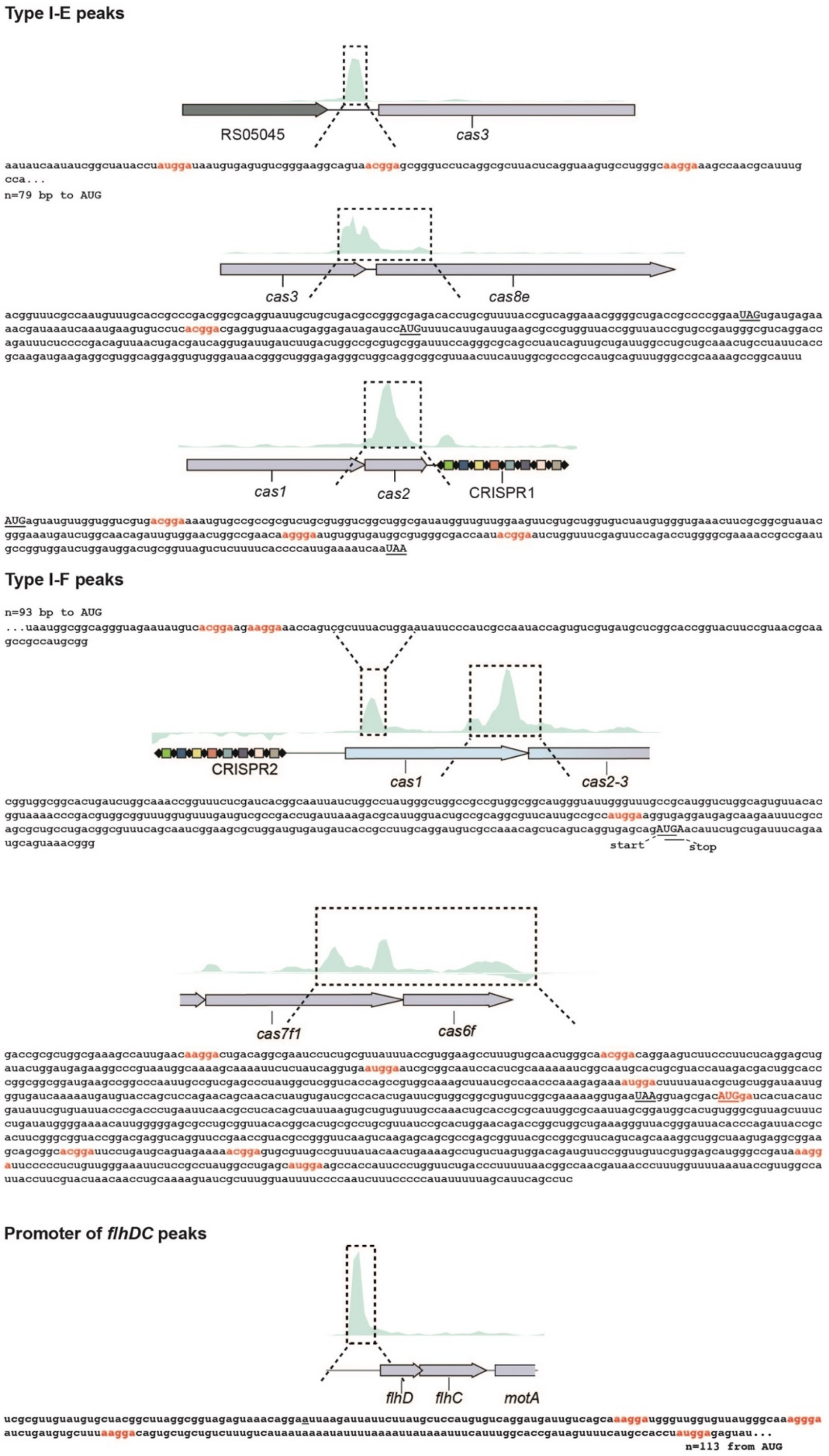

Figure S8. RIP-seq peaks enriched by RsmA in CRISPR-Cas regions. Schematics of the peaks and the sequences present in the CRISPR-Cas operons from **Figure 5C, D** and the promoter region of FlhDC from **Figure 6A**. Consensus RsmA motifs are shown in red, start and stop codons are in uppercase and underlined or the number of nucleotides to the nearest start codon is indicated.

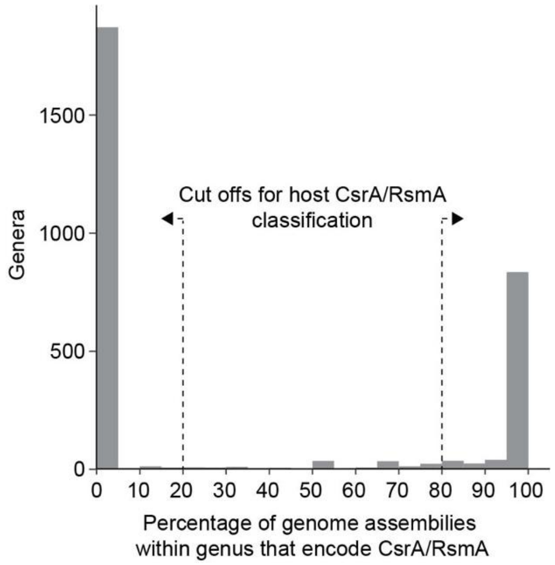

Figure S9. Classification of bacterial genera as CsrA/RsmA hosts. Using the Pfam CsrA Hidden Markov Model (HMM), we determined the proportion of RefSeq bacteria genome assemblies that encode CsrA/RsmA. Each bacterial genera was then classified as either typically encoding CsrA/RsmA (identified in >80% of assemblies), or typically lacking CsrA/RsmA (<20% of assemblies). These classifications were used for inferring whether the hosts of phages (where typically only the host genus is known) encode CsrA/RsmA.

REFERENCES

1. Thomson, N.R., Crow, M.A., McGowan, S.J., Cox, A. and Salmond, G.P. (2000) Biosynthesis of carbapenem antibiotic and prodigiosin pigment in *Serratia* is under quorum sensing control. *Mol Microbiol*, **36**, 539-556.
2. Wilf, N.M., Reid, A.J., Ramsay, J.P., Williamson, N.R., Croucher, N.J., Gatto, L., Hester, S.S., Goulding, D., Barquist, L., Lilley, K.S. *et al.* (2013) RNA-seq reveals the RNA binding proteins, Hfq and RsmA, play various roles in virulence, antibiotic production and genomic flux in *Serratia* sp. ATCC 39006. *BMC Genomics*, **14**, 822.
3. Hampton, H.G., McNeil, M.B., Paterson, T.J., Ney, B., Williamson, N.R., Easingwood, R.A., Bostina, M., Salmond, G.P. and Fineran, P.C. (2016) CRISPR-Cas gene-editing reveals RsmA and RsmC act through FlhDC to repress the SdhE flavinylation factor and control motility and prodigiosin production in *Serratia*. *Microbiology*, **162**, 1047-1058.
4. Smith, L.M., Jackson, S.A., Malone, L.M., Ussher, J.E., Gardner, P.P. and Fineran, P.C. (2021) The Rcs stress response inversely controls surface and CRISPR-Cas adaptive immunity to discriminate plasmids and phages. *Nat Microbiol*, **6**, 162-172.
5. Bell, K.S., Sebaihia, M., Pritchard, L., Holden, M.T., Hyman, L.J., Holeva, M.C., Thomson, N.R., Bentley, S.D., Churcher, L.J., Mungall, K. *et al.* (2004) Genome sequence of the enterobacterial phytopathogen *Erwinia carotovora* subsp. *atroseptica* and characterization of virulence factors. *Proc Natl Acad Sci U S A*, **101**, 11105-11110.
6. Bowden, S.D., Eyres, A., Chung, J.C., Monson, R.E., Thompson, A., Salmond, G.P., Spring, D.R. and Welch, M. (2013) Virulence in *Pectobacterium atrosepticum* is regulated by a coincidence circuit involving quorum sensing and the stress alarmone, (p)ppGpp. *Mol Microbiol*, **90**, 457–471.
7. Thoma, S. and Schobert, M. (2009) An improved Escherichia coli donor strain for diparental mating. *FEMS Microbiol Lett*, **294**, 127-132.
8. Patterson, A.G., Jackson, S.A., Taylor, C., Evans, G.B., Salmond, G.P., Przybilski, R., Staals, R.H. and Fineran, P.C. (2016) Quorum Sensing Controls Adaptive Immunity through the Regulation of Multiple CRISPR-Cas Systems. *Mol Cell*, **64**, 1102-1108.
9. Richter, C., Dy, R.L., McKenzie, R.E., Watson, B.N., Taylor, C., Chang, J.T., McNeil, M.B., Staals, R.H. and Fineran, P.C. (2014) Priming in the Type I-F CRISPR-Cas system triggers strand-independent spacer acquisition, bi-directionally from the primed protospacer. *Nucleic Acids Res*, **42**, 8516-8526.
10. Patterson, A.G., Chang, J.T., Taylor, C. and Fineran, P.C. (2015) Regulation of the Type I-F CRISPR-Cas system by CRP-cAMP and GalM controls spacer acquisition and interference. *Nucleic Acids Res*, **43**, 6038-6048.
11. Jackson, S.A., Birkholz, N., Malone, L.M. and Fineran, P.C. (2019) Imprecise Spacer Acquisition Generates CRISPR-Cas Immune Diversity through Primed Adaptation. *Cell host & microbe*, **25**, 250-260 e254.
12. Gristwood, T., McNeil, M.B., Clulow, J.S., Salmond, G.P. and Fineran, P.C. (2011) PigS and PigP regulate prodigiosin biosynthesis in *Serratia* via differential control of divergent operons, which include predicted transporters of sulfur-containing molecules. *J Bacteriol*, **193**, 1076-1085.
13. Malone, L.M., Warring, S.L., Jackson, S.A., Warnecke, C., Gardner, P.P., Gumy, L.F. and Fineran, P.C. (2020) A jumbo phage that forms a nucleus-like structure evades CRISPR-Cas DNA targeting but is vulnerable to type III RNA-based immunity. *Nat Microbiol*, **5**, 48-55.
14. Vakulskas, C.A., Potts, A.H., Babitzke, P., Ahmer, B.M. and Romeo, T. (2015) Regulation of bacterial virulence by Csr (Rsm) systems. *Microbiol Mol Biol Rev*, **79**, 193-224.
15. Romeo, T. and Babitzke, P. (2018) Global Regulation by CsrA and Its RNA Antagonists. *Microbiol Spectr*, **6**.
